# Supplementary material for: Early–middle Permian Mediterranean gorgonopsian suggests an equatorial origin of therapsids
Source: Nat Commun. 2024 Dec 17;15:10346. doi: 10.1038/s41467-024-54425-5 (PMC11652623; doi:10.1038/s41467-024-54425-5)
Supplement: Supplementary file 1 — Supplementary Information [file 41467_2024_54425_MOESM1_ESM.pdf]

Supplementary Information

Early–middle Permian Mediterranean gorgonopsian suggests an equatorial origin of therapsids

Rafel Matamales-Andreu[1,2]*, Christian F. Kammerer[3], Kenneth D. Angielczyk[4], Tiago R. Simões[5], Eudald Mujal[2,6], Àngel Galobart[2,7] & Josep Fortuny[2]

[1]MUCBO | Museu Balear de Ciències Naturals, FJBS-MBCN, ctra. Palma-Port de Sóller km 30,5, 07100 Sóller, Mallorca, Illes Balears, Spain.

[2]Institut Català de Paleontologia Miquel Crusafont (ICP-CERCA), Universitat Autònoma de Barcelona, Edifici ICTA-ICP, c/ Columnes s/n, Campus de la UAB, 08193 Cerdanyola del Vallès, Barcelona, Catalunya.

[3]North Carolina Museum of Natural Sciences, 11 W. Jones Street, Raleigh, North Carolina 27604 USA.

[4]Field Museum of Natural History, 1400 South Lake Shore Drive, Chicago, Illinois 60605 USA.

[5]Department of Ecology and Evolutionary Biology, Princeton University, Princeton-NJ, 08540, USA.

[6]Staatliches Museum für Naturkunde Stuttgart, Rosenstein 1, 70191 Stuttgart, Germany.

[7]Museu de la Conca Dellà, c/ del Museu 4, 25650 Isona i Conca Dellà, Lleida, Spain.

*Corresponding author: rmatamales@mucbo.org

Contents

Supplementary Note 1: Institutional abbreviations 3

Supplementary Note 2: Detailed description and Supplementary Figs. 1–5 5

Supplementary Note 3: Changes in the phylogeny dataset, Supplementary Table 1 and Supplementary Fig. 6 22

Supplementary References 72

Supplementary Note 1: Institutional abbreviations

AMNH: American Museum of Natural History, New York, USA.

BP: Evolutionary Studies Institute, Johannesburg, South Africa.

CGP/CGS: Council for Geoscience, Pretoria, South Africa.

DA/21: Museu de Mallorca, Palma, Mallorca, Balearic Islands, Spain.

FMNH: Field Museum of Natural History, Chicago, USA.

GMV: Geological Museum of China, Beijing, China.

IGCAGS: Institute of Geology, Chinese Academy of Geological Sciences, Beijing, China.

IVPP: Institute of Vertebrate Paleontology and Paleoanthropology, Beijing, China.

KPM: Vyatka Paleontological Museum, Kirov, Russia.

LfULG RS/SS: Sächsisches Landesamt für Umwelt, Landwirtschaft und Geologie, Freiberg, Germany.

MB.R: Museum für Naturkunde, Berlin, Germany.

MCZ: Museum of Comparative Zoology, Cambridge, USA.

NHCC: National Heritage Conservation Commission, Lusaka, Zambia.

NHMUK: The Natural History Museum, London, United Kingdom.

NMMNH: New Mexico Museum of Natural History and Science, Albuquerque, USA.

NMQR: National Museum, Bloemfontein, South Africa.

PIN: Paleontological Institute of the Russian Academy of Sciences, Moscow, Russia.

ROM: Royal Ontario Museum, Toronto, Canada.

ROZ: Roy Oosthuizen Collection, Iziko South African Museum, Cape Town, South Africa.

SAM: Iziko South African Museum, Cape Town, South Africa.

TM: Ditsong National Museum of Natural History, Pretoria, South Africa.

TMM: Texas Science & Natural History Museum, Austin, USA.

UCMP: University of California Museum of Paleontology, Berkeley, USA.

US: University of Stellenbosch, Stellenbosch, South Africa.

USNM: National Museum of Natural History (Smithsonian Institution), Washington D.C., USA.

Supplementary Note 2: Detailed description and Supplementary Figs. 1–5

Craniomandibular elements

Preserved definite cranial elements consist of a flattened portion of the snout roof, a partial basicranium, and a pterygoid. The snout portion (Supplementary Fig. 1a) is largely made up of the paired nasals. Preservation of the snout is very poor, and because of crushing, it is difficult to even determine whether the exposed bone surface is external or internal. Based on the apparent presence of sculpturing on what is here interpreted as the anterior right nasal, it seems to be external, but this is not certain. The median suture between the nasals is slightly raised. A large, semicircular element on the left side of the snout piece is interpreted as the left maxilla; its surface is extremely poorly preserved, but a rounded thickening near its anterior edge may represent the root of an upper canine. Another very poor section of bone is an amorphous mass at the anterolateral tip of the snout; based on position this could be part of the septomaxilla, but it is too incomplete to say anything about its morphology.

The pterygoid (Supplementary Fig. 1b) is the best-preserved and most informative of the cranial elements. It is nearly complete, missing only the posterior tips of the quadrate rami and part of the anterior margin of the right transverse process. It is exposed in dorsal view, so it is uncertain whether the palatal bosses are preserved. Anteriorly, the pterygoid bears a short (1.7 cm from anterior tip to base of transverse process), attenuate process that would have fit between the palatines. The anterior tip is broken, so it is uncertain whether it would have extended to contact the vomer (inferred to be the plesiomorphic condition in gorgonopsians[1]) or whether such a contact was excluded by a midline suture of the palatines, as in most known gorgonopsians[2,3] (reconstructed as a synapomorphy of the ‘African clade’ of Kammerer & Masyutin[1]). The median

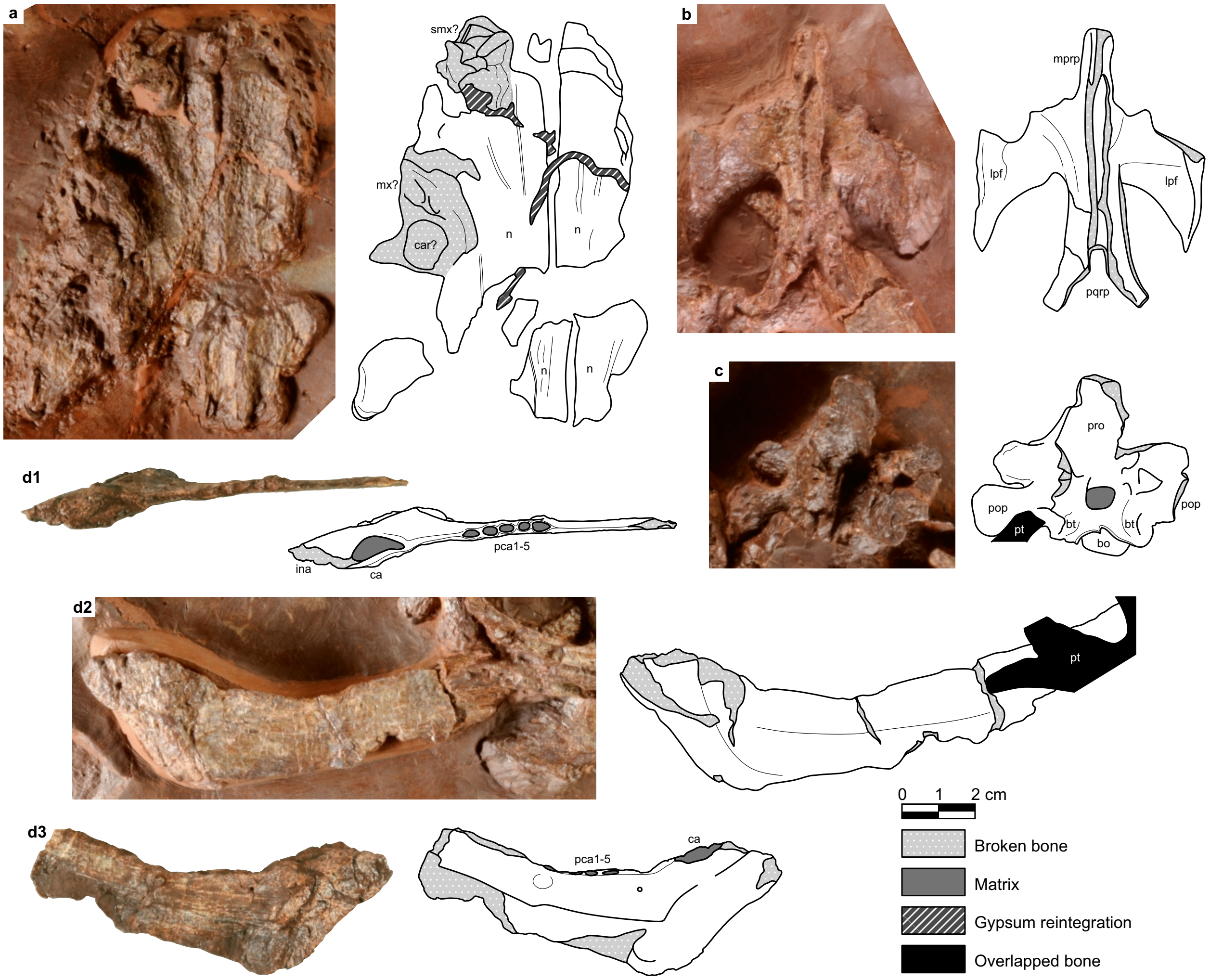

Supplementary Fig. 1. Photographs and interpretative drawings of craniomandibular elements of the gorgonopsian from Mallorca. a, Nasals and possible fragments of left maxilla and septomaxilla, dorsal view. **b**, Pterygoid, dorsal view, **c**, Basicranium, anterior view. **d1**, Left dentary, dorsal view. **d2**, Left dentary, labial view. **d3**, Left dentary, lingual view. Abbreviations: bo = basioccipital, bt = basal tuber, ca = alveolus of canine, car = root of the canine, ina = alveoli of incisors, lpf = lateral pterygoid flange, mprp = median palatal ramus of the pterygoid, mx = maxilla, n = nasal, pca = alveoli of postcanines, pop = paroccipital process (opisthotic), pqrp = pterygoid posterior quadrate rami, pro = prootic, pt = pterygoid, smx = septomaxilla.

septum of the pterygoids is preserved as a low (~0.4 cm dorsoventral height) ridge on the anterior process, which becomes taller (~1.0 cm) beginning at the anterior margin of the transverse process, but this likely reflects incomplete preservation, as this is still quite low compared to that observed in intact skulls (see, *e.g.*, Kemp[4], Bendel *et al.*[5]). Lateral to the median septum, weak depressions are present, which expand anterolaterally towards the anterior margins of the transverse processes. Bounding these depressions laterally are weak ridges connecting the transverse processes to the quadrate rami. Posterior to the median septum is a median facet that would have accommodated the parasphenoid, bounded on either side by weak ridges along the medial edges of the quadrate rami.

The transverse processes of the pterygoid are 'wing'-like, with extremely elongate, attenuate, backswept tips. These are unlike the pterygoids of rubidgeines[6] and related African gorgonopsians (*e.g.*, *Arctops*[7]), in which the lateral edges are thickened, without extensive tapering. However, similar transverse processes are known in earlier-diverging taxa, such as *Cynariops*[5]. The anterior margin of the transverse process bears a prominent notch (best preserved on the left side), which would have accommodated the ectopterygoid. The shape and position of this notch is extremely similar to that of *Cynariops*[5]. The quadrate rami begin to diverge in a relatively anterior position in this specimen, at the level of the tips of the transverse processes. Generally in gorgonopsians, the quadrate rami diverge at a more posterior point on the basicranial girder (*e.g.*, Kemp[4], Bendel *et al.*[5], Kammerer[6–8], Kammerer *et al.*[9]). However, some early-diverging gorgonopsians such as *Eriphostoma* and *Gorgonops* show comparable proportions (see, *e.g.*, Kammerer *et al.*[9]).

The basicranial element (Supplementary Fig. 1c) consists of a fused complex composed minimally of basioccipital, opisthotic, and prootic (the exoccipitals are not exposed, but likely are also included in this complex). Fusion between these braincase elements is common (if not universal)

in adult gorgonopsians[5,10] (but see Araújo *et al.*[11], for delimitations based on a supposed juvenile). The complex is exposed anteroventrally, with a large median cavity where the parabasisphenoid (or parasphenoid-basipresphenoid *sensu* Araújo *et al.*[11]) would have attached. The basioccipital contributions to the basal tubera project posterolaterally. The articular facets that would have connected to the parabasisphenoid are exposed; these are mostly flat, but with a weak median ridge. This morphology of the basal tubera (with the bulbous contributions of the parabasisphenoid overlapping those of the basioccipital ventrally) is observed in many gorgonopsians. The occipital condyle is not fully exposed, but appears to be a single, reniform structure (1.7 cm in transverse width) as is typical of gorgonopsians, rather than the tripartite element of various other therapsid clades (*e.g.*, dinocephalians, anomodonts[12]). The paroccipital process of the opisthotic is relatively short (1.6 cm in length). It is separated from the prootic by a gap of 0.4 cm.

The only preserved mandibular element is the left dentary (Supplementary Fig. 1d1–d3). The bone surface is poorly preserved; some sculpturing and possibly foramina appear to have been present on the external surface of the mandibular symphysis, but it is not possible to provide more specific details. The posterior portion of the dentary, including the coronoid process, is preserved beneath the pterygoid and thus is poorly exposed. What can be seen of the coronoid process demonstrates that it was weakly sloping, forming an angle of roughly 160º relative to the long axis of the dentary. Overall, the dentary is relatively elongate (visible preserved length 14.0 cm, with symphyseal height 4.2 cm from ventral edge to anterior margin of canine alveolus, with ramus height 2.4 cm immediately anterior to pc1 and 2.0 cm immediately posterior to pc5). This morphology differs from that observed in *Aelurosaurus felinus* (NHMUK PV R339) and *Cynariops robustus* (MB.R.999; see[5]), where the dentary is anteroposteriorly shorter with a taller, more sharply sloping

coronoid process, but it is similar to the morphology present in many other gorgonopsians (*e.g.*, '*Delphaciognathus paucidens*', AMNH FARB 5562; see Broom[13]).

The dentary symphysis is angled roughly 120º relative to the long axis of the dentary. Its anterior face is concave in lateral view. This concavity is the result of an anterodorsal projection in front of the canine root that houses the incisors; otherwise, the symphysis sharply but evenly slopes posteroventrally-to-anterodorsally, creating the prominent mentum characteristic of gorgonopsians. This morphology (steep symphysis around the canine root, but inflecting anteriorly around the incisor roots) is present in almost all small gorgonopsians, with the apparent exception of the basal taxon *Nochnitsa* (the lack of an incisor inflection in this taxon is probably due to it having only a weakly sloping dentary symphysis to begin with, like that of basal therocephalians[1]). In larger gorgonopsians, the symphysis is usually massive, with a straight, steeply sloping anterior face lacking an inflection point around the incisors (*e.g.*, in rubidgeines[6]). An articular facet for the splenial is visible on the internal surface of the dentary. The splenial would have contributed to the base of the symphysis and was roughly half the height of the mandibular ramus (facet height 1.0 cm and total height of ramus 2.1 cm at level of pc3).

The incisor alveoli are poorly preserved, but the standard gorgonopsian count of four[10] appears to be present. A single, large canine alveolus is present (1.6 cm in length, 0.5 cm maximum width). The canine root can be discerned in the form of a ridge along the side of the symphysis, immediately anterior to a dorsoventrally elongate depression that would have accommodated the upper canine. The postcanine tooth row is separated from the canine alveolus by a diastema (1.4 cm in length), as is usual for gorgonopsians. Five small (0.3 cm long and 0.2 cm wide) postcanine alveoli are present. The postcanine tooth row is angled anterodorsally-to-posteroventrally, such that the anteriormost tooth is also the most dorsally positioned. This configuration of the dentary

postcanine row is present in all gorgonopsians (with the exception of those lacking multiple postcanines), and is also usual for biarmosuchians (*e.g.*, *Ustia atra*, PIN 4548/155) and therocephalians (*e.g.*, *Glanosuchus macrops*, SAM-PK-11942). Five lower postcanines is a relatively high count for gorgonopsians, but equivalent counts are present in some taxa (*e.g.*, *Aelurosaurus felinus*, NHMUK PV R339), and greater numbers are known in others (*e.g.*, *Nochnitsa geminidens*, KPM 310, with six).

At least four small, isolated teeth (Supplementary Fig. 2a–d) are scattered on the block adjacent to the craniomandibular elements. All appear to be incisors: they are elongate, very weakly recurved, and somewhat spatulate. No striations or facets are present on the enamel surface, but all are serrated, and where both mesial and distal carinae are visible, both show serrations. Given their count, size, and proximity to the dentary, it is possible that these teeth represent the left lower incisors, but this is speculation.

A single isolated canine is preserved (Supplementary Fig. 2e). Although roughly in the same area of the block as the craniomandibular elements, it was found much further from these bones than the incisors and is less likely to have originated in the left dentary (although this cannot be excluded as a possibility). The canine is weakly recurved and blade-like—laterally compressed with mesially and distally serrated edges. It is 1.5 cm wide at its base, and the length of the intact tooth is 4.4 cm (5.3 cm long if including the impression of an additional, missing portion). Serrations run 3.1 cm up the mesial edge of the tooth, suggesting that the entirety of the crown is preserved and the base of this element represents the start of the root. The serrations are very fine, four per millimetre on the better-exposed mesial carina. An indeterminate, splint-like fragment of bone is preserved close to (2.0 cm from) the canine.

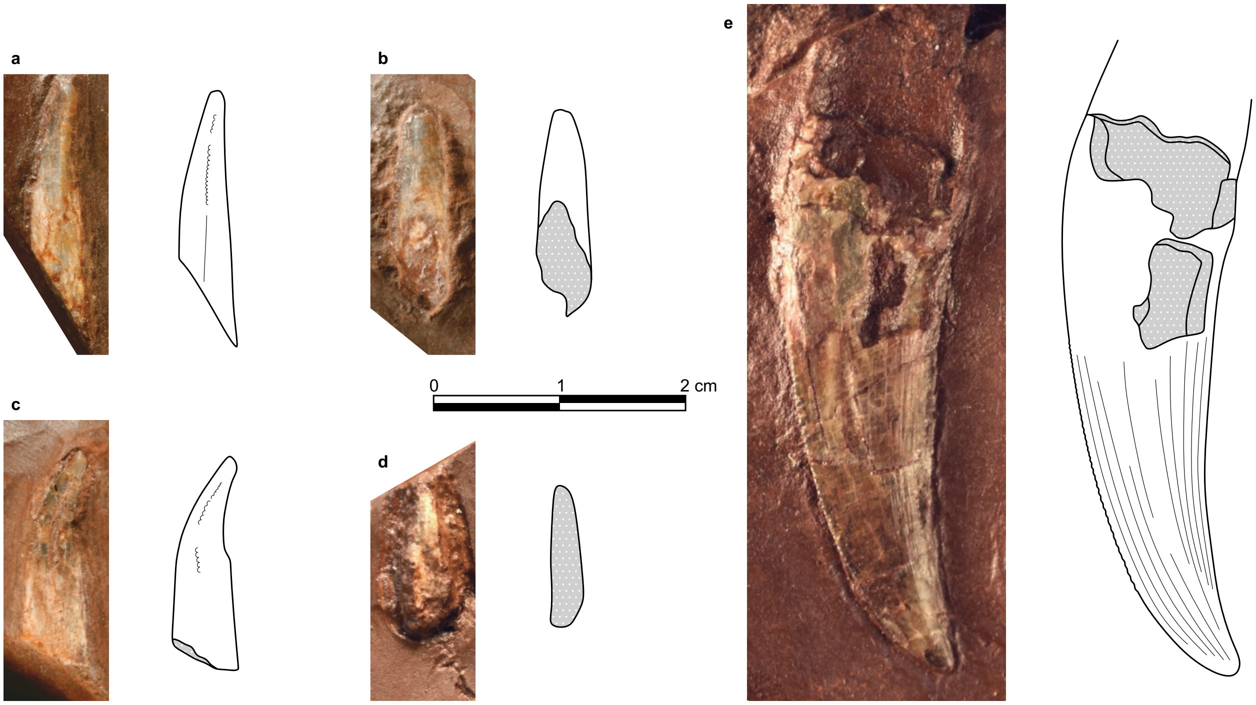

Supplementary Fig. 2. Photographs and interpretative drawings of teeth of the gorgonopsian from Mallorca. a–d, Possible incisors. **e**, Possible upper canine. Colours and patterns as in Supplementary Fig. 1.

A possible additional palatal element is preserved widely separated from the other craniomandibular bones, closer to the tibia. It is a broad, very poorly preserved bone bearing two ridges perpendicular to one another, which bound a depression covering most of its surface (Supplementary Fig. 3a). If part of the skull, this could represent the ectopterygoid or a portion of the palatine (if exposed dorsally), but given the preservation of this element, precise identification is not possible. Near it is another mass of bone, which is an unidentifiable fragment. Another mysterious element is a small (~4.5 cm total length, tip broken off but impression of it present), weakly sinuous bone that may represent a hyoid (Supplementary Fig. 3b). Hyoid elements have not been described in gorgonopsians, but are commonly preserved in therocephalians and

cynodonts, where they are simple, slightly bowed rods. Alternatively, this could represent a rib from the posterior portion of the dorsal series, near the sacrum. Although gorgonopsians do not have true lumbar ribs, their ribs do tend to become proportionally thicker and more curved towards the end of the series.

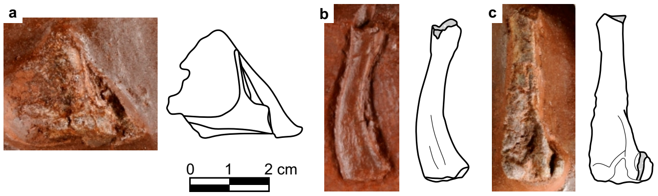

Supplementary Fig. 3. Photographs and interpretative drawings of elements of the gorgonopsian from Mallorca with uncertain identifications, and an element of another animal associated with the studied skeleton. a, Possible ectopterygoid. **b**, Possible hyoid element or posterior dorsal rib. **c**, Distal fragment of a small femur of another animal, posterior view. Colours and patterns as in Supplementary Fig. 1.

<u>Postcranial elements</u>

Preserved axial elements are few, consisting of various isolated ribs and vertebrae plus a large section of the tail. Ten posterior caudal vertebrae are preserved (Supplementary Fig. 4a), split into two articulated groups (the anterior consisting of six and the posterior of four) separated by a gap of 4.5 cm. When collected, this gap area, originally exposed on the outcrop, showed impressions of additional vertebrae, indicating that an additional three or four vertebrae would have been present. All of the articulated vertebrae are very small (0.7–0.9 cm in length) and the posteriormost ones would have been very close to the termination of the series. Tail length variation in Gorgonopsia is poorly understood due to limited material; in complete skeletons (*e.g.*,

Viatkogorgon ivakhnenkoi, PIN 2212/61[14]), between 16 and 19 caudal vertebrae are present, suggesting that the majority of the tail is preserved in this specimen. Two more anterior caudals are preserved in isolation (Supplementary Fig. 4b). The larger of the two (1.5 cm anterior centrum width, 1.1 cm centrum length) is nearly complete (missing only the neural spine) and bears large, posterolaterally oriented transverse processes. The smaller one (1.2 cm anterior centrum width, 1.0 cm centrum length) is more crushed, and only preserves a fragment of the right transverse process.

Adjacent to the cranial elements, two fragmentary, probable cervical vertebrae are preserved. The more complete of the two is exposed in left lateral view (Supplementary Fig. 4c); it has suffered shear and most of the lateral face of the centrum is missing. Identification as a cervical is based on its proportions, it is relatively short and tall (centrum length 2.1 cm, height at anterior face 2.2 cm, height of vertebra at base of neural spine 3.4 cm). The neural spine and prezygapophyses are broken, but the right postzygapophysis is intact and shows that it was largely flat, but angled slightly dorsolaterally at its tip. The other vertebra is a fragment consisting mostly of the neural arch with prezygapophyses (Supplementary Fig. 4d), preserved close to the basicranium. The prezygapophyses are close together, with flat articular facets that are weakly angled dorsolaterally.

Two additional partial vertebrae, of uncertain position, were found widely separated from the other elements, and consist of a distorted centrum (Supplementary Fig. 4e1–e6) and a badly damaged vertebral fragment (mostly centrum), with centrum lengths of 2.3 cm and 1.7 cm, respectively.

Two right dorsal ribs (Supplementary Fig. 4g) are preserved on the underside of the block section containing the dentary, pterygoid, and basicranium. Both were complete as preserved, but one has had its tip split off through the original separation of the blocks. They have been exposed posterior side up. They are indistinguishable from those of other gorgonopsians (see, *e.g.*, Tatarinov[14],

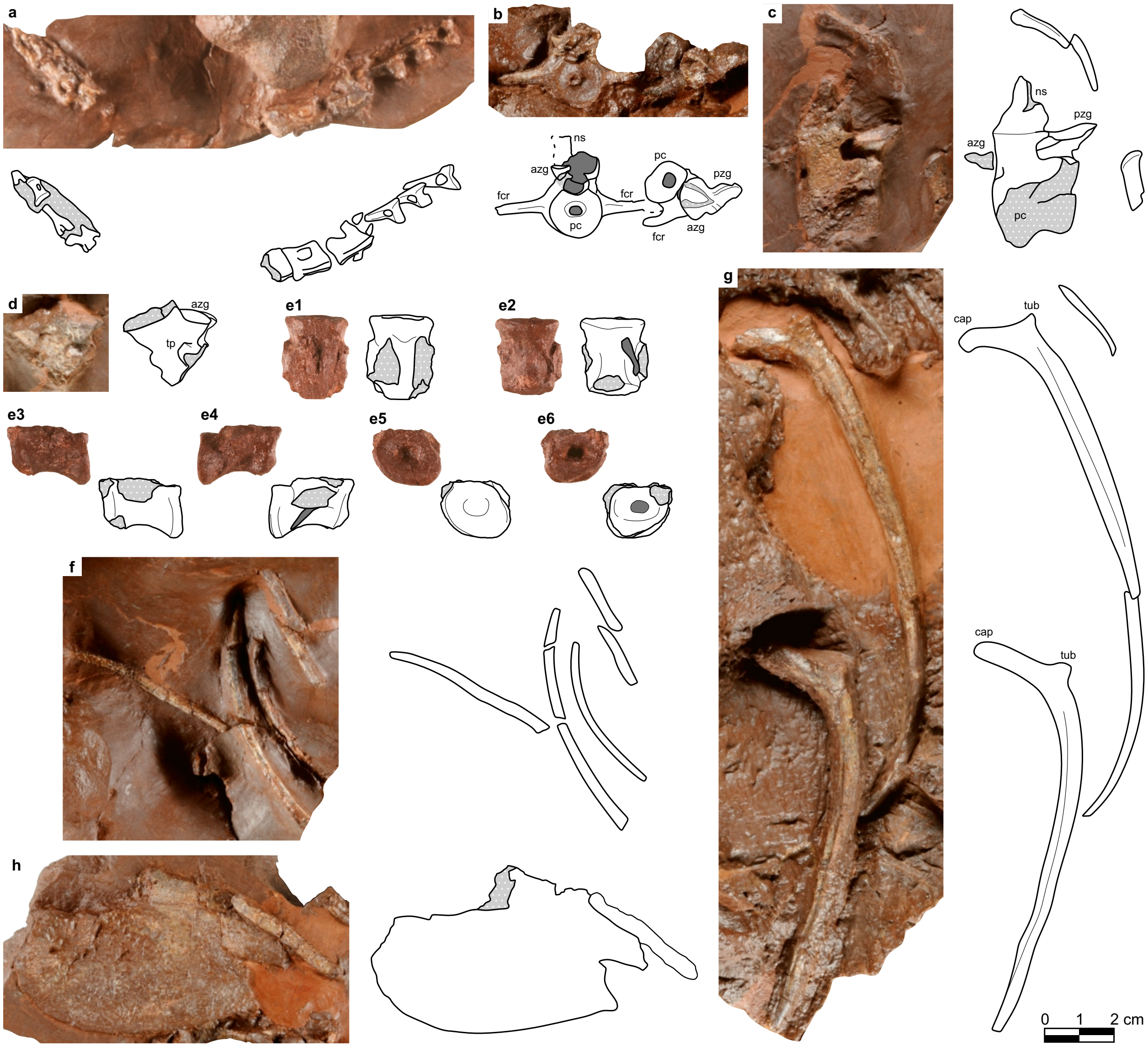

Supplementary Fig. 4. Photographs and interpretative drawings of axial and possible girdle elements of the gorgonopsian from Mallorca. a, Articulated distal caudal vertebrae. **b**, Proximal caudal vertebrae. **c**, Possible cervical vertebra and fragmentary ribs. **d**, Possible cervical vertebral neural arch. **e1**, Vertebral pleurocentrum, dorsal view. **e2**, Vertebral pleurocentrum, ventral view. **e3**, Vertebral pleurocentrum, right lateral view. **e4**, Vertebral pleurocentrum, left lateral view. **e5**, Vertebral pleurocentrum, anterior view. **e6**, Vertebral pleurocentrum, posterior view. **f**, Fragmentary ribs. **g**, Right dorsal ribs. **h**, Possible part of the pelvis (illium or ischion). Abbreviations: azg = prezygapophysis, cap = capitulum, fcr = fused caudal ribs, ns = neural spine,

pc = pleurocentrum, pzg = postzygapophysis, tp = transverse process, tub = tubercle. Colours and patterns as in Supplementary Fig. 1.

Gebauer[15]), with bicipital heads and weakly curved bodies. The total length of the complete rib along its curvature is 17.5 cm. The tips of at least four additional ribs (Supplementary Fig. 4f) are preserved near the tarsals and fibula.

Several well-preserved limb elements are present, but few, if any, girdle elements are preserved. A large (preserved dimensions 7.9 × 4.5 cm), laminar bone fragment near the caudal series probably represents part of the pelvic girdle (either the ilium or ischium) (Supplementary Fig. 4h), but it is too incomplete and poorly preserved for definite identification.

The left femur has been prepared out in the round (Supplementary Fig. 5a1–a6). It is 14.9 cm in total length. Proximally it is 3.9 cm wide, 2.0 cm at mid-length (5.0 cm circumference), and 4.1 cm wide distally. The head is well-ossified and not confluent with the greater trochanter, which is a low, curved structure extending 4.5 cm down the anterolateral margin of the shaft. The distal condyles are massive, but with shallow intertrochanteric and popliteal fossae. In general, this element is similar to that of other small-to-mid-sized gorgonopsians, showing sinuous curvature[3,15,16] as opposed to the more straight-shafted morphology in larger taxa[3,17].

The left fibula (Supplementary Fig. 5b) was preserved disarticulated from but in contact with the femur. It is a very narrow, elongate element whose shaft is weakly bowed. The proximal and distal tips are greatly expanded where it would have articulated with the femur/tibia and tarsals, respectively. The proximal articular surface is convex, whereas the distal articular surface is nearly

flat. The fibula is 11.5 cm in total length, and mediolaterally is 1.9 cm wide at its proximal end, 0.7 cm at wide at mid-length, and 2.0 cm wide at its distal end.

The left tibia is exposed in posterior view (Supplementary Fig. 5c), and shows the typical gorgonopsian morphology[3,15,16], being robust and strongly bowed. The weak cnemial crest is visible at the anteromedial edge of the exposed bone. The tibia is 10.2 cm in total length, and mediolaterally is 3.8 cm wide at its proximal end, 1.7 cm wide at mid-length, and 2.3 cm wide at its distal end.

An additional element preserved in the same general area as the tibia appears to represent the distal half of another femur (Supplementary Fig. 3c). However, this bone is much smaller (length of preserved portion 4.1 cm) than the other femur, with a much more gracile morphology. Identification as a femur is based on general shape and presence of well-ossified anterior and posterior trochanters at the inferred distal tip. This is the only element in the block that seems to belong to an individual other than the gorgonopsian. Damage to this bone makes taxonomic identification difficult, but it is consistent with the morphology of small reptiles[18].

A variety of pedal elements are scattered across the block; they represent a mixture of left and right elements, suggesting a complex history of disarticulation. Although incomplete, enough of the tarsals, metatarsals (mt), and phalanges (ph) are preserved to recognise that this animal had a typical gorgonopsian pes[3,19,20].

The only definitely preserved tarsal elements are the left calcaneum and astragalus. Both are typical for gorgonopsians, and previous descriptions of these elements (*e.g.*, Colbert[16], Sidor[20], Schaeffer[21]) could also apply to this specimen. The calcaneum (Supplementary Fig. 5d) has the standard primitive therapsid condition, being disc-shaped and notched medially. Its dimensions

are 3.5 × 2.8 cm. The astragalus (Supplementary Fig. 5e) is a multilobate element with bulbous surfaces for articulation with the tibia and fibula and a channel for the perforating artery. Its dimensions are 2.4 × 2.3 cm.

The metatarsal preserved closest to the craniomandibular elements has been prepared out in the round (Supplementary Fig. 5f1–f6). It measures 2.8 cm in total length, and mediolaterally it is 1.6 cm wide at its proximal end, 0.7 cm wide at mid-length, and 1.5 cm wide at its distal end. It is very thin dorsoventrally (0.2 cm at mid-length), suggesting taphonomic compression. Considering the great degree of expansion of its proximal and distal ends, and their asymmetry, it is identified as the right mt5. In general morphology, it is similar to the equivalent element described in the Zambian gorgonopsian NHCC LB1073[20], with dorsally oriented proximal and ventrally oriented distal articular facets, but it differs in some minor details. The proximal articular facets are more asymmetrical than in NHCC LB1073, with the medial portion wider (1.0 cm) than the lateral (0.5 cm). In proximal view, the surface is attenuate at both the medial and lateral edges (it is only attenuate laterally in NHCC LB1073), but the lateral portion is thicker, with the median ridge dividing the proximolateral portions into equal dorsal and ventral portions (in NHCC LB1073, the dorsal portion is dominant). Although these differences may be partially influenced by taphonomy, that is unlikely to explain all of them, particularly considering that the thicker proximal surface would seem to be opposite from the observed direction of compression for the rest of this element.

Two additional metatarsals were preserved in isolation closer to the femur/fibula, which are identified as the left mt4 and mt3. The mt4 (exposed ventral side up) (Supplementary Fig. 5g) is the longest preserved metatarsal (total length 3.2 cm, mediolateral width of proximal end 1.0 cm, mid-length 0.5 cm, and distal end 1.5 cm). Its proximal end is notably more compact than in mt5, and it bears a dorsomedial facet for articulation with mt3. The mt3 (Supplementary Fig. 5h) is

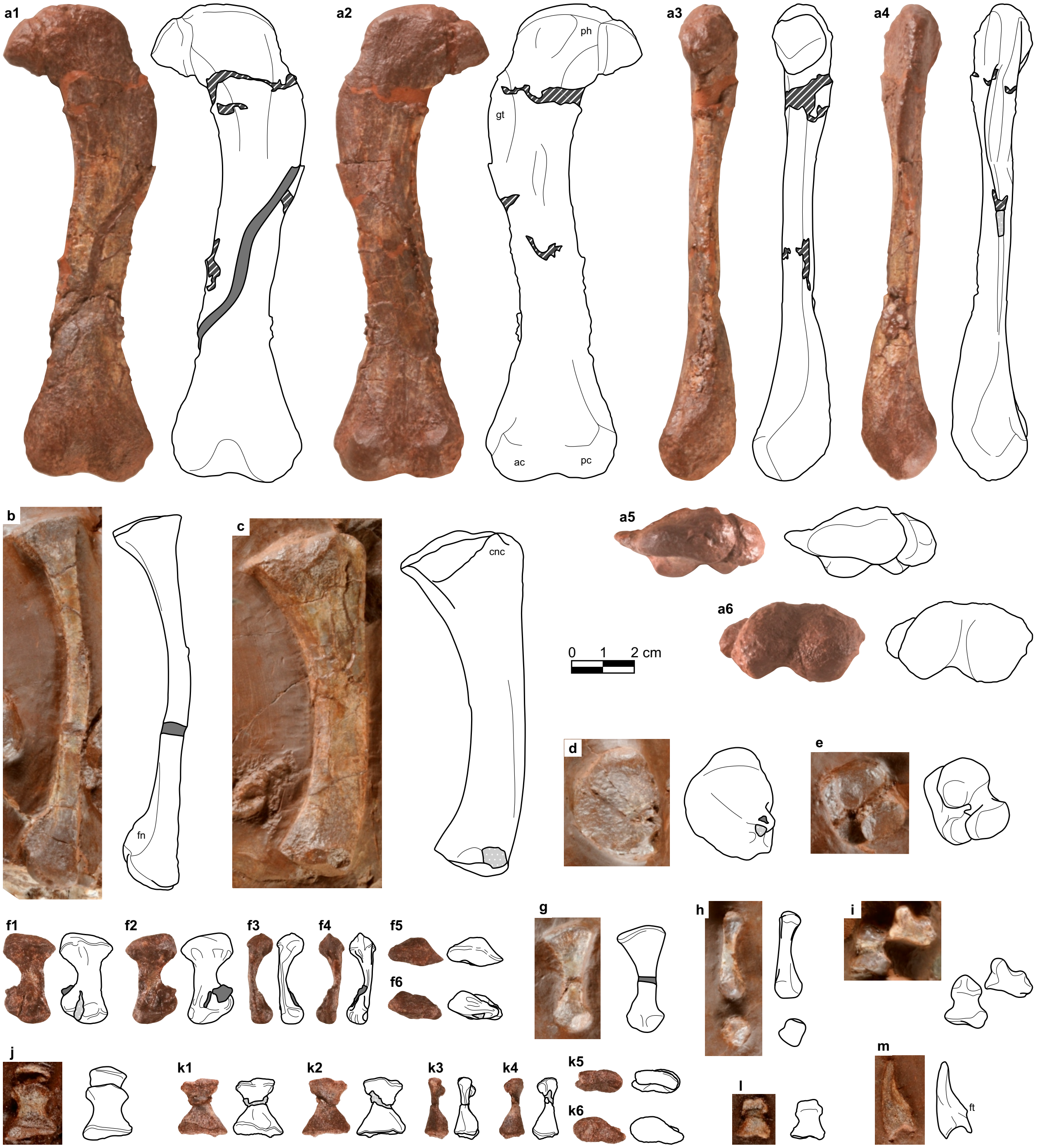

Supplementary Fig. 5. Photographs and interpretative drawings of hindlimb and pedal elements of the gorgonopsian from Mallorca. a1, Left femur, anterior view. **a2**, Left femur, posterior view. **a3**, Left femur, medial view. **a4**, Left femur, lateral view. **a5**, Left femur, proximal view. **a6**, Left femur, distal view. **b**, Left fibula, anterior view. **c**, Left tibia, posterior view. **d**, Left

calcaneum, dorsal view. **e**, Left astragalus, dorsal view. **f1**, Right fifth metatarsal, dorsal view. **f2**, Right fifth metatarsal, ventral view. **f3**, Right fifth metatarsal, lateral view. **f4**, Right fifth metatarsal, medial view. **f5**, Right fifth metatarsal, distal view. **f6**, Right fifth metatarsal, proximal view. **g**, Left fourth metatarsal, ventral view. **h**, Left third distal tarsal and left third metatarsal, lateral view. **i**, Right first metatarsal and first phalanx of right digit I, ventral view. **j**, First and second phalanges of right digit IV, dorsal view. **k1**, First phalanx of right digit V, dorsal view. **k2**, First phalanx of right digit V, ventral view. **k3**, First phalanx of right digit V, lateral view. **k4**, First phalanx of right digit V, medial view. **k5**, First phalanx of right digit V, distal view. **k6**, First phalanx of right digit V, proximal view. **l**, Penultimate phalanx, dorsal view. **m**, Ungual (terminal phalanx), lateral or medial view. Abbreviations: ac = anterior condyle, cnc = cnemial crest, fn = fibular notch, ft = flexor tubercle, gt = greater trochanter, pc = posterior condyle, ph = proximal head of femur. Colours and patterns as in Supplementary Fig. 1.

preserved on its side; its identity is inferred from being morphologically nearly identical to that of the mt4, except smaller (total length 2.6 cm, other measures not possible to take precisely because of angle of exposure). Close (0.8 cm) to the proximal tip of mt3 is a small, cuboidal bone, which may represent the third distal tarsal (Supplementary Fig. 5h).

Two pedal elements are preserved immediately adjacent to the basicranium, consisting of the right mt1 and ph1 of digit I (Supplementary Fig. 5i). The metatarsal is 1.6 cm in total length, and mediolaterally it is 1.5 cm wide at its proximal end, 0.8 cm wide at mid-length, and 1.0 cm wide at its distal end. The phalanx is 1.3 cm in total length, and mediolaterally it is 1.0 cm wide at its proximal end, 0.5 cm wide at mid-length, and 0.8 cm wide at its distal end. Although disarticulated, these elements are still in contact, suggesting that disarticulation occurred late in the depositional

history of the specimen, probably shortly before burial. Both are exposed ventrally, showing the morphology of the distal articular facet of the metatarsal and proximal articular facet of the phalanx. The proximal articular facet of the metatarsal is not visible, indicating that it was dorsally oriented, as in other early synapsids[22]. Unlike the other metatarsals, mt1 is short, thick, and highly asymmetrical, with an enlarged medial but reduced lateral lobe at its proximal end. The distal articular region is narrow and also asymmetrical, with the medial facet for articulation with the phalanx wider than the lateral. This asymmetry is reflected in the morphology of the distal portion of the phalanx: the medial condyle is mediolaterally broader and flatter, whereas the lateral condyle is shorter but more bulbous. The articular head of the phalanx bears a weak, distally-oriented protrusion, as in other gorgonopsians[23].

An additional three phalanges are preserved near the small femur. Two of these appear to represent ph1 and ph2 of the right digit IV, exposed dorsally (Supplementary Fig. 5j). The second phalanx of this set is discoidal, indicating that these must be from digit III, IV, or V[19]. Ph1 is highly asymmetrical proximally, with a pointed expansion medially (ruling out digit III by comparison to other gorgonopsians[3,20]). The phalanges of digits IV and V are difficult to distinguish in isolation, but the third phalanx preserved in this part of the block (see below) is more consistent with being the ph1 of digit V, because it is larger and more elongate. The proximal articular facet of the digit IV ph1 is dorsally oriented, with a ventrally expanded ‘lip’ (more prominent medially). The phalanx is roughly ‘dumbbell’-shaped, but with the proximal expansions greater than the distal. The distal articular facet is ventrally oriented. The discoidal phalanx is only semi-disarticulated, exposing its proximal articular facet. This facet is weakly concave, with greater curvature laterally than medially, following the edge of the distal ph1. The ph1 is 1.7 cm in total length, and mesiodistally is 1.4 cm wide at its proximal end, 0.8 cm wide at mid-length, and 1.2 cm wide at

its distal end. The discoidal ph2 is 0.2 cm long and 1.2 cm wide. The third phalanx (here interpreted as the ph1 of digit IV) (Supplementary Fig. 5k1–k6) is very similar to the other ph1, but with slightly different proportions (1.9 cm total length, mediolateral width of proximal end 1.5 cm, 0.6 cm at mid-length, and 1.2 cm at distal end).

An additional dorsally-exposed phalanx (Supplementary Fig. 5l) is preserved in an isolated position roughly equidistant between the tibia and femur. Based on size and general morphology, it is definitely a penultimate phalanx, but its digital identity is uncertain; for digits III–V these phalanges are very difficult to distinguish in isolation. Like the other non-discoidal phalanges, it is a 'dumbbell'-shaped element that is broader proximally than distally (total length 1.2 cm, mediolateral width 1.1 cm distally, 0.6 cm at mid-length, and 0.8 cm distally). At its proximal end, it has an expanded lateral lobe protruding proximally; its distal end is trapezoidal, with a broad trochlea for articulation with the ungual.

A single ungual phalanx (Supplementary Fig. 5m) is preserved on the underside of the block containing the tibia. Known gorgonopsian unguals are generally conservative in morphology, and it is consistent with previously described examples (*e.g.*, Sidor[20]). The articular facet is prominently cupped, and a well-developed flexor tubercle is visible on the exposed proximoventral portion of the bone. The ungual is elongate and only weakly curved (ungual tip extends at an angle of roughly 20º from the dorsal and ventral edges of the articular facet).

Supplementary Note 3: Changes in the phylogeny dataset, Supplementary Table 1 and Supplementary Fig. 6

Character changes

27. 'The position of the posterior border of the choana: close to the incisor (0), far behind the incisor (1).' This character is original to the analysis of Liu *et al*.[24] and is coded as '0' for all non-therapsid synapsids, *Raranimus*, and *Biarmosuchus*. This is a vague character, but the position of the posterior border of the choana in all synapsids is substantially posterior to the incisors. In *Dimetrodon limbatus*, the posterior border of the choana is posterior to both maxillary caniniforms, for example. The morphology of the choana in *Biarmosuchus* is extremely similar to that of biarmosuchians coded as '1' in previous versions of the analysis (*e.g., Hipposaurus, Herpetoskylax*). Unlike the characters below, where there is some underlying morphological variation present in the idea behind the character permitting retention with alteration, there is no apparent variation in this character. All taxa preserving the choana have been coded as '1' for this character in the current matrix.

64. 'Upper incisors: much larger (0) or roughly equivalent in size to postcanines (1).' This character is inherently problematic in terms of determining what constitutes 'much' larger, but is also difficult to code because of substantial size variation between tooth positions. For example, the anteriormost premaxillary tooth in *Haptodus* (based on ROM 43606) is substantially larger than any post-caniniform maxillary tooth, but subsequent premaxillary teeth are increasingly smaller, with the second and third tooth positions being slightly larger than most post-caniniform teeth and the fourth and fifth tooth positions being smaller than most post-caniniform teeth. Herein,

this character is changed to ‘Upper incisors: maximum size greater than that of largest postcanine (0) or roughly equivalent in size to largest postcanine (1)’ to at least address this latter issue.

74. Formerly ‘Interpterygoid vacuity: long (0) or short (1)’, changed to ‘Interpterygoid vacuity: transversely broad (0) or narrow and slit-like (1)’. This character as described by Amson & Laurin[26] is redundant with character 72 (‘Pterygoid at level of posterior edge of transverse flange’), with the character states ‘far from sagittal plane, leaving the interpterygoid vacuity posteriorly opened (0)’ and ‘interpterygoid vacuity closed or constricted posteriorly by median flange (1)’ being necessarily equivalent to its states 0 and 1 (the third state of character 72, ‘quadrate processes of pterygoid medially appressed (2)’, results in the absence of an interpterygoid vacuity, making character 74 inapplicable). There is variation in morphology of the interpterygoid vacuity that is not directly correlated with its posterior constriction, *i.e.*, whether it is expansive (as in some non-therapsid synapsids) or reduced to a narrow slit (as in most therapsids that retain interpterygoid vacuities at maturity, which are mostly narrow and slit-like [dicynodonts, which are not represented in this data set, are an exception, but the enlarged interpterygoid vacuity of dicynodonts is a derived feature related to the major changes in pterygoid architecture in that clade and not a shared feature with non-therapsid synapsids]). Numerous taxa in the data set of [25] were coded as ‘2’ or ‘1&2’ for this character, despite the fact that no state 2 exists in their formulation. These taxa have all been recoded in the current analysis.

76. Formerly ‘Epipterygoid ventral plate: large, part of basicranium (0) or small, excluded from basicranium (1)’, changed to ‘Epipterygoid ventral plate: situated high on the cranium dorsal to the primary palate (0), situated low on the cranium, roughly at the same plane of the primary palate (1).’ The previous formulation of this character[25] is problematic. The example they figure of a ‘small’ epipterygoid footplate (that of the gorgonopsian *Arctops willistoni* [identified as

‘*Lycaenops angusticeps*’ therein, but see[7]) is equal in anteroposterior dimensions to that of the taxon identified as having a ‘large’ epipterygoid footplate (*Dimetrodon limbatus*). The surface area of the epipterygoid footplate in *Arctops* is also only slightly less than that of *Dimetrodon*, and well within the range of non-therapsid synapsid taxa coded as ‘0’ for this character. However, the nature of the contribution of the epipterygoid footplate to the basicranium does show important variability, as figured by [25]. In non-therapsid synapsids (*e.g.*, *Dimetrodon*, they also depict this morphology for *Haptodus* and *Tetraceratops*; fig. 4A–C in[25]), the epipterygoid columella is relatively short, such that the footplate is located dorsal to the primary palate, and the footplate is angled posterodorsally-to-anteroventrally on the sharply-angled dorsal surface of the basicranial ramus of the pterygoid. In therapsids (*e.g.*, *Syodon*, *Arctops*; fig. 4D, E in[25]), the epipterygoid columella is relatively long, such that the footplate is at a similar level as the primary palate, and the footplate is nearly horizontal, lying dorsal to a similarly horizontally-oriented basicranial pterygoid ramus.

<u>New taxa coded (with list of specimens used for coding)</u>

Patelosaurus saxonicus (LfULG RS 14752)

Palaeohatteria longicaudata (LfULG RS 14754-14755 [counterpart and part of syntype], SS 13004-13005, SS 13305-13310, SS 13315-13317, SS 13124-13125, SS 13535)

Cutleria wilmarthi (USNM 22099)

Archaeosyodon praeventor (PIN 1758/3 [holotype], PIN 1758/95, PIN 1758/293, PIN 1758/328)

Tapinocaninus pamelae (NMQR 2985, NMQR 2987 [holotype])

Ulemica efremovi (PIN 2793/1 [holotype])

Viatkogorgon ivakhnenkoi (PIN 2212/6 [holotype])

Suchogorgon golubevi (PIN 4548/1 [holotype], PIN 4548/10)

Eriphostoma microdon (AMNH FARB 5524 [holotype], BP/1/7275, SAM-PK-K11164)

Arctops willistoni (BP/1/698, CGS 319, CGS RMS 63, UCMP 42701)

Lycosuchus vanderrieti (CGS C60, MB.R.995, US D173)

<u>Existing taxa recoded</u>

Haptodus garnettensis (ROM 29872, ROM 30099, ROM 43601, ROM 43602, ROM 43604, ROM 43606)

2. **?**, previously coded as 0. All known specimens of *Haptodus* are extremely compressed. It is likely that the snout was taller than wide in this taxon, as in other three-dimensionally-preserved sphenacodont skulls, but this is not determinable in the known material—in ROM 43606, which is laterally compressed, the snout appears taller than wide, but ROM 43602, which is dorsoventrally compressed, the snout appears wider than tall.

21. **?**, previously coded as 0. No specimens of *Haptodus* preserve the zygomatic arch and quadrate intact and in life position.

27. **1**, previously coded as 0.

42. **?**, previously coded as 0. Articulated pterygoids are not preserved in any specimens of *Haptodus*. Both pterygoids are preserved in disarticulation in ROM 43602, and it can be seen that they would not have had a broad contact anterior to the basicranium, but whether they were broadly or narrowly separated in life position is indeterminate.

46. **?**, previously coded as 0. No three-dimensionally-preserved crania or articulated occiputs are known for *Haptodus*, making confident coding of this character impossible.

47. **?**, previously coded as 0. In the specimens of *Haptodus* with the best-preserved opisthotics (ROM 43602 and ROM 43604), those elements are still incomplete and badly crushed, making accurate determination of the orientation of the paroccipital processes impossible.

48. **?**, previously coded as 0. No specimens of *Haptodus* preserve the quadrate in articulation with both the paroccipital process and the squamosal.

50. **-**, previously coded as ?. *Haptodus* lacks a distinct dentary caniniform tooth (or teeth), making this character inapplicable.

55. **0**, previously coded as ?. Although *Haptodus* (like *Tetraceratops*) lacks the enlarged reflected lamina of the angular present in sphenacodontids and elaborated further in therapsids, the notch associated with the ancestral angular keel is evident in ROM 43601 and is in posterior position.

66. **-**, previously coded as ?. No distinct dentary caniniform tooth is present in *Haptodus*, so this character is inapplicable.

72. **1**, previously coded as 0. Although not in articulation, the well-preserved and ventrally well-exposed left pterygoid of ROM 43602 shows a short flange of the pterygoid medial to the main basicranial ramus of this element that appears comparable in morphology to that of *Tetraceratops*, in which a medial flange partially bounds the posterior margin of the interpterygoid vacuity.

75. **1**, previously coded as 0. The quadrate of *Haptodus* is smaller relative to skull size than that of *Dimetrodon* and *Sphenacodon*, and although it is not preserved in association with an intact cranium for direct comparison, the well-exposed quadrate in ROM 43604 can be interpreted as making up less than half of the skull by comparison with the associated dentary and squamosal.

76. **?**, previously coded as 0. No specimens of *Haptodus* preserve a complete epipterygoid with exposed footplate.

77. **?**, previously coded as 0. This is a problematic character generally, as it is difficult to define what constitutes ‘loosely’ *vs.* ‘firmly’ sutured, but it cannot be coded for *Haptodus* as no specimens preserve an articulated occiput. The failure to find articulated occipital elements of *Haptodus* cannot be used as the basis for considering them ‘loosely’ sutured, because all known *Haptodus* material is crushed and disarticulated to varying degrees, with extensive observed disarticulation of presumably “firmly” sutured elements of the skull roof and palate.

Sphenacodon ferox (FMNH UC 1218, NMMNH P-55367, UCMP 34226)

27. **1**, previously coded as 0.

44. **1**, previously coded as ?. The ectopterygoid is well preserved in NMMNH P-55367 and shows no evidence of dentition.

59. **?**, previously coded as 0. The articular is insufficiently preserved to code this character in all mandibular specimens of *S. ferox*. A dorsal process is present in *S. ferocior*[26], but in the current version of the analysis, OTUs have been restricted to single species.

Dimetrodon milleri (MCZ 1365 [holotype], MCZ 1367)

27. **1**, previously coded as 0.

64. **0**, previously coded as 1. The anteriormost premaxillary teeth in MCZ 1365 exceed any of the postcaniniform teeth in apicobasal length.

Secodontosaurus obtusidens (AMNH FARB 4007 [holotype], AMNH FARB 4091, MCZ 1124)

27. **?**, previously coded as 0. The anterior palate is not exposed in any specimens of *Secodontosaurus*.

28. **?**, previously coded as 0. The anterior palate is not exposed in any specimens of *Secodontosaurus*.

31. **?**, previously coded as 0. CT slices of MCZ 1124[27] permit determination of some aspects of vomerine morphology in this specimen (allowing coding of characters 29, 30,

and 32), but due to extreme compression of the skull the relative positions of the snout elements have been distorted, and characters 31 and 33 cannot be coded confidently.

33. **?**, previously coded as 0.

35. **?**, previously coded as 0. The palatines are only preserved in MCZ 1124 and are obscured and their positions in CT data are distorted due to compression of the skull.

47. **?**, previously coded as 0. All known *Secodontosaurus* specimens preserving the opisthotic are so badly crushed as to prohibit shape determination of the paroccipital process.

48. **?**, previously coded as 0. The quadrate in *Secodontosaurus* probably has the typical sphenacodontid articulation (primarily with the paroccipital process), but in the only specimen with a well-preserved quadrate (MCZ 1124), the opisthotic is barely exposed and only quadrate-squamosal contact is visible.

66. **-**, previously coded as ?. No distinct dentary caniniform tooth is present in *Secodontosaurus* so this character is inapplicable.

72. **1**, previously coded as 0. MCZ 1124 shows a short flange of the pterygoid medial to the main basicranial ramus of this element that appears comparable in morphology to that of *Tetraceratops*, in which a medial flange partially bounds the posterior margin of the interpterygoid vacuity. This interpretation is also shown in the idealized cranial reconstruction of *Secodontosaurus* by Reisz *et al*.[27], which was informed primarily by MCZ 1124.

78. **1**, previously coded as 0. Minimal variation in anterior dentary tooth size is observed in *Secodontosaurus*, and such that exists can be attributed largely to eruption history.

Dimetrodon limbatus (AMNH FARB 4636, FMNH UC 1001, MCZ 1347)

27. **1**, previously coded as 0.

Dimetrodon grandis (FMNH UC 1002, USNM 8635)

7. **0**, previously coded as ?. As illustrated by Brink *et al*.[28], the septomaxilla in FMNH UC 1002 is restricted to the naris.

27. **1**, previously coded as 0.

55. **0**, previously coded as ?. Enough of the reflected lamina is preserved in USNM 8635 to show that its notch would be closer to the articular than the dentary.

Tetraceratops insignis (AMNH FARB 4526 [holotype])

6. **1**, previously coded as 0. This is a very vague character, but the snout length of AMNH FARB 4526 is comparable or shorter than that of the therapsid taxa coded for this character (preorbital and postorbital regions of skull subequal).

10. **0**, previously coded as ?. The orbital rim is damaged in AMNH FARB 4526, but enough of the dorsal margin is preserved on the left side to say that it was not thickened with some confidence.

11. **0**, previously coded as ?. The temporal region is damaged in AMNH FARB 4526, so the exact area of the temporal fenestra is unknown, but the total length of the temporal

region is known (based on the right side and proportions of the basicranium) and it is physically impossible to fit a temporal fenestra even larger than the proportionally enormous orbit into this space.

12. **?**, previously coded as 1/2. Determination of the adductor musculature attachment site in AMNH FARB 4526 has been the center of a large amount of controversy, which has heavily influenced interpretations of whether *Tetraceratops* is the earliest therapsid. The crux of the issue is whether the bar-like element in this specimen represents the lateral skull roof (postorbital and squamosal; as argued by Laurin & Reisz[29,30] and Amson & Laurin[25]) or the zygomatic arch (jugal and squamosal; as argued by Conrad & Sidor[31] and Spindler[32]). This author's (CFK) interpretation of the element generally accords with the latter in considering it most likely zygomatic; however, AMNH FARB 4526 is so poorly preserved that definite identification of this thin bone fragment is probably impossible without information from additional specimens.

14. **?**, previously coded as 0. The supratemporal portion of the postorbital in AMNH FARB 4526, if preserved at all (which is arguable, see above), is not complete enough to determine its posterior terminus.

16. **?**, previously coded as 0. The complete sutural boundaries of the postfrontal are indeterminable in AMNH FARB 4526.

41. **0**, previously coded as ?. A large interpterygoid vacuity, similar to that of coded non-therapsid sphenacodont taxa, broadly separates the basicranial rami of the pterygoids in AMNH FARB 4526.

43. **0**, previously coded as ?. Exact sutural boundaries between the pterygoid and parabasisphenoid are not visible in AMNH FARB 4526, which is probably why this character was not coded in previous versions of the analysis. However, clear in AMNH FARB 4526, and is illustrated in the reconstructions of Laurin & Reisz[30] and Spindler[32], is that the basicranial portion of the pterygoid is both greatly dorsoventrally expanded and elevated dorsally above its palatal portions, as in, *e.g., Dimetrodon*. This necessitates the basipterygoid articulation, obscured though it may be, being dorsal to the primary palate.

44. **?**, previously coded as 1. The ectopterygoid is not visible in AMNH FARB 4526.

50. **-**, previously coded as ?. No distinct dentary caniniform tooth (or teeth) can be discerned in AMNH FARB 4526, making this character inapplicable.

51. **0**, previously coded as ?. The right dentary is sufficiently preserved to show that a low coronoid eminence, not a freestanding coronoid process, is present in AMNH FARB 4526.

55. **0**, previously coded as ?. Like *Haptodus, Tetraceratops* has only a weak angular keel, but there is a distinct notch on the right angular in AMNH FARB 4526 near the articular.

56. **0**, previously coded as ?. The lateral surface of the right angular is preserved in AMNH FARB 4526. It exhibits an autapomorphic ventral thickening and rugosity, but no ridges or fossae. This character specifically refers to the ornamentation of the reflected lamina in therapsids, but as the character is phrased only with regards to the angular surface and not the reflected lamina, it can still be coded for *Tetraceratops*.

66. **-**, previously coded as ?. No distinct dentary caniniform tooth is present in *Tetraceratops,* so this character is inapplicable.

67. **?**, previously coded as 0. None of the maxillary caniniform teeth in AMNH FARB 4526 have preserved crowns, so the presence of heels is indeterminable.

69. **?**, previously coded as 1. The upper postcaniniform count of AMNH FARB 4526 is uncertain due to poor preservation. As many as 15 dentary postcaniniform teeth appear to be present, suggesting the maxillary tooth count could greatly exceed the observed seven tooth positions.

73. **?**, previously coded as 1. This character's coding is also contingent on identification of the rod-like element discussed in Character 12. Also, even if this element is the postorbital and squamosal, it is extremely questionable whether the amount of muscular attachment on the squamosal depicted by Amson & Laurin[25] can be discerned.

74. **0**, previously coded as 1. The dimensions of the interpterygoid vacuity in AMNH FARB 4526 are only slightly less than those of *Haptodus*, which was coded 0.

76. **0**, previously coded as 1.

77. ?, previously coded as 1. Damage to the back of the skull makes confident identification of most of the occipital elements impossible in AMNH FARB 4526.

Raranimus dashankouensis (IVPP V 15424 [holotype])

2. **?**, previously coded as 0. The snout is incomplete and laterally compressed in IVPP V 15424, making its proportions uncertain.

4. **0**, previously coded as 1. The dorsal process of the premaxilla reaches to the point of the anterior upper canine in IVPP V 15424 but does not appear to extend posterior to it, and certainly does not extend beyond the posterior upper canine.

8. **?**, previously coded as 2. The identification of the prefrontal of IVPP V 15424 by Liu *et al*.[24] and Duhamel *et al*.[33] is highly dubious, and more likely represents a broken fragment of the nasal.

10. **?**, previously coded as 0. The orbits are not preserved in IVPP V 15424.

27. **1**, previously coded as 0.

33. **1**, previously coded as 0. The CT reconstruction of the vomer by Duhamel *et al*.[33] shows the choanal portion to be displaced ventrally relative to the post-choanal portion.

79. **?**, previously coded as 0. No postcanines are sufficiently well preserved in IVPP V15424 to permit determination of their crown morphology.

Biarmosuchus tener (PIN 1758/1, PIN 1758/2 [holotype], PIN 1758/7, PIN 1758/8, PIN 1758/85)

5. **0**, previously coded as 1. Premaxillary morphology in *Biarmosuchus* specimens is somewhat variable. Ivakhnenko[34] considered the nominal species *Biarmosuchus tener* (holotype PIN 1758/2), *Biarmosaurus antecessor* (holotype PIN 1758/7), and *Eotitanosuchus olsoni* (holotype PIN 1758/1) to represent growth stages (of increasing size, respectively) of a single biospecies, a conclusion agreed with here. In cases of polymorphisms with probable ontogenetic influence, codings herein have been based on the largest specimen, PIN 1758/1. In PIN 1758/2 the premaxillary alveolar margin is

upturned, in PIN 1758/7 and PIN 1758/8 it is nearly horizontal, and in PIN 1758/1 it is essentially identical to that of IVPP V 15424. The latter was considered ‘downturned’ by Liu *et al*.[25]; this is somewhat problematic because the involved angles are unclear. In IVPP V 15424, the left premaxilla is slightly downturned but the right is nearly horizontal (although this may be due to distortion), and in both cases the downturn is substantially less marked than in sphenacodontids. With this noted, to keep consistency with the formulation of the character by Liu *et al*.[24], *Raranimus* is retained as state 0, but this necessitates coding *Biarmosuchus* the same.

16. **1**, previously coded as 0. Although not as extreme as in later therapsids, a posteromedial curvature of the postfrontal is visible in some *Biarmosuchus* specimens (*e.g.*, PIN 1758/85).

27. **1**, previously coded as 0.

29. **1**, previously coded as ?. The vomer is well exposed and clearly unpaired in PIN 1758/1 and PIN 1758/85.

30. **2**, previously coded as 1. The vomer in *Biarmosuchus* (*e.g.*, PIN 1758/1, PIN 1758/85) is widest anteriorly and the choanal portion is progressively constricted posteriorly, becoming very narrow anterior to the post-choanal portion.

31. **0**, previously coded as ?. In PIN 1758/85, the contact between the choanal and postchoanal portions of the vomer are exposed and relatively undistorted, showing that it is broad and flat.

32. **0**, previously coded as ?. The vomer of *Biarmosuchus* (*e.g.*, PIN 1758/1, PIN 1758/85) has a weakly convex ventral surface for most of its choanal length, becoming flatter anteriorly.

34. **1**, previously coded as 0. The palatines in PIN 1758/1 extend for nearly half the length of the choana.

35. **0**, previously coded as ?. Separation of the palatines ventrally is evident in PIN 1758/85.

43. **1**, previously coded as ?. In all specimens of *Biarmosuchus* preserving the basicranium, the basipterygoid articulation is in a relatively ventral position, at a similar level as the primary palate.

52. **1**, previously coded as 0. The dentaries are poorly preserved in most *Biarmosuchus* specimens, but an incised posterior margin of the dentary appears to be present on the right side of PIN 1758/2.

62. **1**, previously coded as ?. Intermeshing of at least the anterior incisors is evident in PIN 1758/2.

66. **0**, previously coded as ?. A precanine embayment of the choana accommodating the lower canine is visible in, *e.g.*, PIN 1758/1.

68. **0**, previously coded as 1. No diastema is present between the upper canine alveolus and postcanine tooth row in *Biarmosuchus*.

74. **-**, previously coded as 2. Closure of the interpterygoid vacuity in *Biarmosuchus* makes this character inapplicable.

78. **1**, previously coded as 0. The lower incisors of *Biarmosuchus* are subequal in size.

Hipposaurus boonstrai (BP/1/5411, CGS WB 123, SAM-PK-8950 [holotype], SAM-PK-9081)

20. **0**, previously coded as ?. The zygomatic arch lacks bosses in all known specimens of *Hipposaurus* that preserve this element (*e.g.*, SAM-PK-9081).

22. **0**, previously coded as ?. The subtemporal zygoma is well preserved anteriorly in SAM-PK-9081, showing that the zygomatic ramus of the squamosal does not reach the level of the postorbital bar.

28. **0**, previously coded as ?. The vomerine process of the premaxilla is relatively short, comparable to that seen in non-therapsid synapsids, in specimens of *Hipposaurus* exposing this morphology (*e.g.*, BP/1/5411).

29. **1**, previously coded as ?. The vomer is unpaired in specimens of *Hipposaurus* preserving this element (*e.g.*, BP/1/5411, SAM-PK-9081).

30. **0**, previously coded as 1. The choanal portion of the vomer in *Hipposaurus* (*e.g.*, BP/1/5411, SAM-PK-9081) is nearly parallel-sided, it is not substantially expanded at mid-length.

34. **1**, previously coded as ?. In BP/1/5411 and CGS WB 123, the anterior portions of the palatines are elongate, forming nearly half of the lateral margin of the choana.

35. **0**, previously coded as ?. Separation of the palatines by the vomer is visible in BP/1/5411.

52. **1**, previously coded as 0/1. The posterior margin of the dentary is strongly incised in all specimens of *Hipposaurus* with a well-preserved jaw (*e.g.*, CGS WB 123); its apparent absence in some specimens is an artifact of poor preservation.

57. **1**, previously coded as ?. A surangular ridge is present in, *e.g.*, SAM-PK-8950.

66. **0**, previously coded as ?. A precanine embayment of the choana accommodating the lower canine is visible in, *e.g.*, BP/1/5411.

73. **1**, previously coded as ?. Extensive muscular attachment on the squamosal within the temporal fenestra is visible in, *e.g.*, SAM-PK-8950.

74. **-**, previously coded as 2. Closure of the interpterygoid vacuity in *Hipposaurus* makes this character inapplicable.

76. **1**, previously coded as ?. The right epipterygoid is well preserved and exposed in SAM-PK-9081, showing that it had an extremely anteroposteriorly narrow, elongate columella and horizontal footplate at a similar level as the palatal portion of the pterygoid.

78. **1**, previously coded as 0. The lower incisors of *Hipposaurus* are subequal in size.

Herpetoskylax hopsoni (BP/1/3924, CGP/1/67 [holotype])

13. **1**, previously coded as 0. The anteroposterior width of the postorbital bar in CGP/1/67 is ~42% of its dorsoventral height.

14. **1**, previously coded as ?. The posterodorsal corner of the temporal fenestra is composed of postorbital in BP/1/3924.

21. **0**, previously coded as 0/1. The zygomatic arch of *Herpetoskylax* arcs such that its mid-section is dorsal to the postcanine tooth row, as is also the case in some other biarmosuchians (*e.g., Hipposaurus*) and members of other therapsid clades (*e.g.,* most scylacosaurid therocephalians), but in doing so it does not completely expose the quadrate-quadratojugal complex in the same manner as occurs in anomodonts.

31. **1**, previously coded as ?. The vomer in CGP/1/67 constricts to form a sharp median ridge at the contact between its choanal and post-choanal portions.

73. **1**, previously coded as ?. Extensive muscular attachment on the squamosal within the temporal fenestra is visible in CGP/1/67.

74. **-**, previously coded as 2. Closure of the interpterygoid vacuity in *Herpetoskylax* makes this character inapplicable.

75. **1**, previously coded as ?. The quadrate in CGP/1/67 is relatively small compared to that of, *e.g.*, *Dimetrodon*.

78. **1**, previously coded as 0. The lower incisors of *Herpetoskylax* are subequal in size.

Lycaenodon longiceps (NHMUK PV R5700 [holotype])

1. **1**, previously coded as 0. The dorsal margin of the snout is nearly horizontal in NHMUK PV R5700, the only known specimen of *Lycaenodon*.

2. **1**, previously coded as 0. The snout in NHMUK PV R5700 is roughly as wide as it is tall at tip.

11. **?**, previously coded as 0. Intact temporal fenestrae are not preserved in NHMUK PV R5700.

12. **?**, previously coded as 0. Only the right postorbital bar is preserved in NHMUK PV R5700 and its posterior edge is broken, so origination area of the adductor musculature is indeterminable.

13. **1**, previously coded as 0. Although its posterior margin is incomplete, the preserved portion of the postorbital bar in NHMUK PV R5700 is ~50% of its height, more than sufficient to code *Lycaenodon* as '1' for this character, and indicating that it could have been even broader in life.

16. **?**, previously coded as 1. The posterior portion of the postfrontal is not preserved in NHMUK PV R5700.

17. **?**, previously coded as 2. The pineal foramen is not preserved in NHMUK PV R5700.

36. **?**, previously coded as 1. No palatal dentition is preserved in NHMUK PV R5700.

37. **?**, previously coded as 0. No palatal dentition is preserved in NHMUK PV R5700.

38. **?**, previously coded as 0. No palatal dentition is preserved in NHMUK PV R5700.

39. **?**, previously coded as 1. The transverse process of the pterygoid is not preserved in NHMUK PV R5700.

40. **?**, previously coded as 0. The transverse process of the pterygoid is not preserved in NHMUK PV R5700.

41. **?**, previously coded as 1. The basicranial ramus of the pterygoid is not preserved in NHMUK PV R5700.

42. **?**, previously coded as 1. The basicranial ramus of the pterygoid is not preserved in NHMUK PV R5700.

44. **?**, previously coded as 1. The ectopterygoid is not preserved in NHMUK PV R5700.

63. **?**, previously coded as 0. No incisor crowns are preserved in NHMUK PV R5700.

64. **?**, previously coded as 1. Only the incisor roots in cross-section are preserved in NHMUK PV R5700, which is not adequate for determining relative sizes of the teeth.

67. **?**, previously coded as 0. The upper canines of NHMUK PV R5700 are broken off at base, so it cannot be determined whether heels were present.

78. **?**, previously coded as 0. No dentary is preserved in NHMUK PV R5700 so this character cannot be coded.

Lemurosaurus pricei (BP/1/816 [holotype], NMQR 1702)

4. **0**, previously coded as ?. Although sutural boundaries in *Lemurosaurus* are difficult to discern, a clear mid-nasal suture anterior to the canine region is visible in NMQR 1702, and the apparent posterior tip of the dorsal process of the premaxilla can be differentiated from the surrounding bone in BP/1/816.

5. **1**, previously coded as ?. The premaxillary alveolar margin is subhorizontal in both BP/1/816 and NMQR 1702.

13. **1**, previously coded as 0. The anteroposterior width of the postorbital bar in NMQR 1702 is ~57% of its height.

15. **1**, previously coded as 0. A small boss is present at the dorsal edge of the postorbital bar in NMQR 1702.

29. **1**, previously coded as ?. The vomer is unpaired in NMQR 1702.

35. **0**, previously coded as ?. The palatines are separated by the vomer medially in NMQR 1702.

38. **0**, previously coded as 1. A well-developed tooth row on the transverse process of the pterygoid is present in BP/1/816. In the considerably larger (and inferred as more mature) specimen NMQR 1702, there are fewer teeth on the transverse process but at least a few are retained medially.

43. **2**, previously coded as ?. The basipterygoid articulation is visible ventrally in NMQR 1702.

61. **0**, previously coded as ?. Five upper incisors are present in BP/1/816 and NMQR 1702.

64. **0**, previously coded as 1. The upper incisors of BP/1/816 and NMQR 1702 are considerably larger (in basal diameter and apicobasal width) than the upper postcanines.

66. **0**, previously coded as ?. The lower canine of NMQR 1702 fits into a precanine embayment of the choana.

72. **1**, previously coded as 2. A narrow interpterygoid vacuity is present between the pterygoid basicranial rami just posterior to the transverse processes in NMQR 1702; they are not completely appressed.

73. **1**, previously coded as ?. Extensive muscular attachment on the squamosal within the temporal fenestra is visible in NMQR 1702.

75. **1**, previously coded as ?. The quadrate in NMQR 1702 is relatively small compared to that of, *e.g.*, *Dimetrodon*.

Proburnetia viatkensis (PIN 2216/1 [holotype])

6. **0**, previously coded as 1. The length of the snout in PIN 2216/1 is comparable to that of *Hipposaurus* (which is coded as “0”) and exceeds the length of the post-orbital region.

13. **1**, previously coded as 0. The anteroposterior width of the postorbital bar in PIN 2216/1 is ~68% of its height.

29. **1**, previously coded as ?. The vomer of PIN 2216/1 is unpaired.

33. **0**, previously coded as ?. The choanal and post-choanal portions of the vomer are at a similar level in PIN 2216/1.

51. **0**, previously coded as ?. No free-standing coronoid process is present in PIN 2216/1.

61. **0**, previously coded as ?. Examination of the original blocks containing PIN 2216/1 indicate that *Proburnetia* has five upper incisors.

66. **0**, previously coded as ?. The lower canine of PIN 2216/1 fits into a precanine embayment of the choana.

68. **0**, previously coded as ?. No diastema is present between the upper canine and postcanine tooth row in PIN 2216/1.

71. **0**, previously coded as ?. The upper postcanine and incisor tooth rows are not confluent in PIN 2216/1.

74. **-**, previously coded as 2. Closure of the interpterygoid vacuity in *Proburnetia* makes this character inapplicable.

75. **1**, previously coded as ?. The quadrate in PIN 2216/1 is relatively small compared to that of, *e.g.*, *Dimetrodon*.

78. **1**, previously coded as ?. The lower incisors of *Proburnetia* are subequal in size.

Burnetia mirabilis (NHMUK PV R5697)

3. **0**, previously coded as ?. The external nares are situated close to the anterior margin of the premaxilla in NHMUK PV R5697.

5. **2**, previously coded as ?. The premaxillary alveolar margin in NHMUK PV R5697 is strongly upturned, to a degree comparable to that of anteosaurids.

11. **0**, previously coded as 1. The orbit of NHMUK PV R5697 is proportionally smaller than in most non-burnetiine biarmosuchians, but is still slightly anteroposteriorly wider than the temporal fenestra.

13. **1**, previously coded as 0. The anteroposterior width of the postorbital bar in NHMUK PV R5697 is equal to its height.

24. **1**, previously coded as ?. Although the skull roof in NHMUK PV R5697 is heavily pachyostosed, the sutural boundaries of the preparietal can be discerned.

29. **1**, previously coded as ?. The vomer of NHMUK PV R5697 is unpaired.

33. **0**, previously coded as ?. The choanal and post-choanal portions of the vomer are at a similar level in NHMUK PV R5697.

38. **?**, previously coded as 1. The ventral surface of the transverse process of the pterygoid is so badly eroded in NHMUK PV R5697 that it is not possible to tell whether teeth are present.

43. **2**, previously coded as ?. The basipterygoid articulation is visible ventrally in NHMUK PV R5697.

45. **1**, previously coded as ?. The limits of the postparietal can be roughly discerned on the occiput of NHMUK PV R5697, and appears similar to that known in other burnetiids.

46. **2**, previously coded as 1. The occipital plate of NHMUK PV R5697 is strongly angled anteroventrally.

68. **0**, previously coded as ?. No diastema is present between the upper canine and postcanine tooth row in NHMUK PV R5697.

69. **1**, previously coded as ?. The exact number of upper postcanines in NHMUK PV R5697 is uncertain because of damage, but five partial teeth are preserved in the left maxilla and

two alveoli, with very limited space posterior to them that could conceivably accommodate many additional teeth.

74. **-**, previously coded as 2. Closure of the interpterygoid vacuity in *Burnetia* makes this character inapplicable.

Syodon biarmicum (PIN 157/2, PIN 157/684)

13. **0**, previously coded as 1. The maximum anteroposterior width of the postorbital bar in PIN 157/2 is ~23% of its height (minimum width is ~14% of height).

16. **1**, previously coded as 0. In PIN 157/2, as in most therapsids, the postfrontal extends posteromedially, with a posterior process between the postorbital (laterally), frontal (anteromedially), and parietal (posteromedially).

35. **0**, previously coded as 1. An anterior process of the pterygoids contacts the vomer and divides the palatines in PIN 157/2.

38. **0**, previously coded as ?. One definite tooth is present on the left transverse process of the pterygoid in PIN 157/2. More fragmentary *Syodon* specimens show more extensive tooth rows on this structure; in PIN 157/684, the right transverse process of the pterygoid bears five teeth.

50. **1**, previously coded as 0. The mandibular ramus of PIN 157/2 is constricted posterior to the canine.

53. **1**, previously coded as 0. Contra[35,36], there is no evidence of a coronoid bone in anteosaurs. The medial surface of the mandible of PIN 157/2 is well preserved with all of

its elements intact; there is no evidence of a discrete facet for a missing coronoid. The reconstructions of Orlov[37], showing the coronoid region to be occupied by an anterior process of the surangular in anteosaurs, appear to be accurate based on personal (CFK) examination of the specimens in question.

62. **?**, previously coded as 2. The incisors are too damaged in all known specimens of *Syodon* to determine whether they were intermeshing.

64. **1**, previously coded as 0. The preserved incisors (*e.g.*, left I4) of PIN 157/2 are comparable in size or smaller than the postcanines (which bear expanded, fungiform crowns). The cross-sections of the broken more anterior incisors are also within the size range of the postcanines.

68. **1**, previously coded as 0. A lengthy diastema (equivalent to the length of roughly three postcanine positions) separates the canine and postcanine tooth row in PIN 157/2.

74. **-**, previously coded as 2. Closure of the interpterygoid vacuity in *Syodon* makes this character inapplicable.

76. **1**, previously coded as ?. The epipterygoid in PIN 157/2 has an elongate columella placing the footplate at a relatively ventral position, at the level of the primary palate.

77. **1**, previously coded as ?. The occiput of PIN 157/2 shows no sign of disarticulation between basicranial and dermal components.

78. **1**, previously coded as 0. The lower incisors of *Syodon* are subequal in size.

Titanophoneus potens (PIN 157/1 [holotype], PIN 157/3)

13. **0**, previously coded as 1. The anteroposterior length of the postorbital bar in PIN 157/1 is ~24% of its height. In the larger and presumed more mature specimen PIN 157/3, in which this region is pachyostosed, the postorbital bar is thicker, but it is still only roughly 1/3 of its height, insufficient to be coded as ‘1’.

16. **1**, previously coded as 0. In PIN 157/1, the postfrontal extends posteromedially, with a posterior process between the postorbital (laterally), frontal (anteromedially), and parietal (posteromedially). The same appears to be true of PIN 157/3, although heavy pachyostosis of the skull roof in this specimen makes precise determination of sutural boundaries more uncertain.

17. **1**, previously coded as 2. A chimney-like pineal boss is present in PIN 157/1, but this appears to be related to immaturity; in the larger and presumed more mature PIN 157/3, pachyostosis of the skull roof has resulted in the pineal boss becoming a diffuse swelling that is not distinctly raised above the surrounding bone.

37. **0**, previously coded as 1. No teeth are visible on the palatal portion of the pterygoid in PIN 157/1, but this region is damaged; in PIN 157/3, well-developed clusters of palatal teeth are present on both pterygoids.

50. **1**, previously coded as 0. The mandibular ramus of PIN 157/1 is constricted posterior to the canine.

53. **1**, previously coded as 0. As noted for *Syodon*, there is no evidence of a coronoid in *Titanophoneus*.

59. **0**, previously coded as ?. The well-preserved right articular region of PIN 157/1 shows that a dorsal process of the articular is absent in *Titanophoneus*.

74. **-**, previously coded as 2. Closure of the interpterygoid vacuity in *Titanophoneus* makes this character inapplicable.

78. **1**, previously coded as 0. The lower incisors of *Titanophoneus* vary in size, but there is not a single distinctly enlarged tooth: i1–3 are all roughly equivalent in size and i4 is substantially smaller than the others.

Sinophoneus yumenensis (GMV 1601 [holotype], IVPP V 18117, IVPP V 18119, IVPP V 18120, IVPP V 18123, IVPP V 18124)

Previous versions of this analysis[24,25,28] employed the anteosaurian OTU '*Stenocybus*'. Kammerer[38] argued that the holotype of '*Stenocybus acidentatus*' (IGCAGS V 361) represents a very small juvenile specimen of the co-occurring anteosaurid *Sinophoneus yumenensis* (at the time known only from the holotype, GMV 1601). Liu[39] described numerous additional specimens of Dashankou anteosaurids covering a range of sizes intermediate between IGCAGS V 361 and GMV 160, bridging the morphological gaps between them and supporting the conclusion that these represent the growth series of a single species. As such, here the '*Stenocybus*' OTU has been deleted and replaced with one for *Sinophoneus yumenensis*, with priority for coding based on larger (presumed more mature) specimens.

2. **1**, previously coded as 0. Snout proportions vary with ontogeny in *Sinophoneus* (and also related to type and degree of taphonomic distortion). In the smallest, inferred juvenile

specimens, such as the holotype of ‘*Stenocybus acidentatus*’, the snout is much taller than wide, in larger specimens (*e.g.*, IVPP V 18119), the snout is roughly as tall as wide, and in the largest specimen (GMV 1601) the snout is slightly wider than tall (measured at level of upper canine), but the skull appears to have suffered some dorsoventral compression, which would exaggerate its proportional width, so it is coded as ‘1’ here.

3. **1**, previously coded as 0. Position of the external narial opening appears to be an ontogenetically variable character in anteosaurids, being terminal in the smallest specimens (IGCAGS V 361) and moving progressively further back over the course of growth. Retraction of the nares in GMV 1601 is comparable to that of *Syodon* and *Titanophoneus*.

11. **1**, previously coded as 0. The proportionally enormous orbit of ICCAGS V 361 is a juvenile feature; larger specimens of *Sinophoneus* show the typical amniote allometry of proportionally smaller orbital size (and in this taxon, proportionally larger temporal fenestra size).

16. **1**, previously coded as 0. An elongate posterior process of the postfrontal is particularly evident in IVPP V 18120.

26. **0**, previously coded as ?. The occiput is sufficiently well preserved in IVPP V 18120 to show that there is a ventral tabular contact with the paroccipital process of the opisthotic.

27. **1**, previously coded as ?. The choana in GMV 1601 has a posterior border medial to the postcanine tooth row.

28. **2**, previously coded as ?. Although somewhat poorly preserved, the anterior palatal region of GMV 1601 appears to show the usual anteosaurian morphology of the vomer abutting the main body of the premaxilla, without a distinct premaxillary vomerine process.

30. **1**, previously coded as ?. The choanal portion of the vomer in GMV 1601 is widest at mid-length. Given that its edges are damaged, it likely would have been even broader in this region when intact.

31. **1**, previously coded as ?. The contact between the choanal and post-choanal portions of the vomer in GMV 1601 is broad, without a median ridge.

32. **1**, previously coded as ?. Even though damaged, it is evident that the vomer in GMV 1601 is most raised at its edges and concave medially.

33. **0**, previously coded as ?. The contact between the choanal and post-choanal portions of the vomer in GMV 1601 occurs at the same level.

35. **0**, previously coded as ?. Separation of the palatines by the post-choanal plate of the vomer is evident in GMV 1601.

36. **1**, previously coded as ?. Palatine dentition in *Sinophoneus* is restricted to a reniform boss, visible in GMV 1601 and IVPP V 18123.

39. **1**, previously coded as ?. The transverse process of the pterygoid is clearly located at the anterior edge of the orbit in GMV 1601.

43. **1**, previously coded as ?. The basipterygoid articulation in *Sinophoneus* is dorsal to the pterygoid, as seen in, *e.g.*, IVPP V 18123.

44. **1**, previously coded as ?. No teeth are visible on the ectopterygoid in all specimens of *Sinophoneus* exposing this element (*e.g.*, GMV 1601, IVPP V 18123).

48. **1**, previously coded as ?. Roughly equal contributions of the squamosal and paroccipital process of the opisthotic to the quadrate margin are visible in IVPP V 18120.

51. **0**, previously coded as ?. No free-standing coronoid process of the dentary is present in *Sinophoneus*, as can be seen in, *e.g.*, IVPP V 18117, IVPP V 18124.

52. **1**, previously coded as 0. A strongly incised posterior margin of the dentary is evident in IVPP V 18120.

57. **0**, previously coded as ?. No surangular ridge is visible in, *e.g.*, IVPP V 18117, IVPP V 18120.

59. **0**, previously coded as ?. No dorsal process is present in *Sinophoneus* specimens preserving the articular (*e.g.*, IVPP V 18119, IVPP V 18123).

66. **1**, previously coded as ?. A fossa roofed by the premaxilla and maxilla is present in GMV 1601 to accommodate the lower canine.

68. **0**, previously coded as ?. No diastema separates the upper canine and postcanine tooth rows in *Sinophoneus* (*e.g.*, IVPP V 18117).

72. **2**, previously coded as ?. As in other anteosaurids, the quadrate rami of the pterygoid are tightly appressed immediately posterior to the transverse processes in *Sinophoneus* (*e.g.*, IVPP V 18117).

73. **1**, previously coded as ?. The squamosal of *Sinophoneus* has an extensive attachment site for jaw musculature within the temporal fenestra (*e.g.*, GMV 1601, IVPP V 18120).

74. **-**, previously coded as 2. Closure of the interpterygoid vacuity in *Sinophoneus* makes this character inapplicable.

75. **1**, previously coded as ?. The quadrate in *Sinophoneus* specimens (*e.g.*, IVPP V 18120) is relatively small compared to that of, *e.g.*, *Dimetrodon*.

76. **1**, previously coded as ?. The epipterygoid is well preserved in IVPP V 18119 and exhibits a long columella with a ventral footplate overlying the pterygoid at the level of the primary palate.

77. **1**, previously coded as ?. The elements making up the occiput are tightly articulated in *Sinophoneus* specimens (*e.g.*, IVPP V 18120).

Styracocephalus platyrhynchus (SAM-PK-8936 [holotype], SAM-PK-K8071)

11. **0**, previously coded as ?. Likely related to heavy pachyostosis of the temporal region, the lateral exposure of the temporal fenestra is small relative to the total skull size of *Styracocephalus*, and is smaller than the orbit (although the orbit is also relatively small compared to that of most other taxa coded in this analysis).

20. **1**, previously coded as 0. A large boss is present on the zygomatic arch in SAM-PK-8936.

24. **0**, previously coded as ?. Although skull roof sutures in *Styracocephalus* specimens are largely obliterated through pachyostosis, the eroded area around the pineal foramen in SAM-PK-K8071 shows median sutures of the parietals but no evidence of a preparietal.

25. **?**, previously coded as 1. Although likely absent as in other therapsids, because of pachyostosis of the skull roof, sutures cannot be determined in the area where a supratemporal would occur if present in *Styracocephalus*.

58. **?**, previously coded as 0. The medial face of the mandible in SAM-PK-8936 is too badly damaged to determine whether this foramen was present.

74. **-**, previously coded as 2. Closure of the interpterygoid vacuity in *Styracocephalus* makes this character inapplicable.

78. **1**, previously coded as 0. The lower incisors of *Styracocephalus* are subequal in size.

Jonkeria truculenta (FMNH UC 1511, ROZ.B96, SAM-PK-4343, SAM-PK-11884, SAM-PK-12030, TM 212 [holotype])

7. **1**, previously coded as 2. The septomaxilla of *Jonkeria* only extends outside of the naris as a short splint (visible in FMNH UC 1511), it does not have an elongate posterior process.

13. **1**, previously coded as 0. The anteroposterior width of the postorbital bar in SAM-PK-434 is 2/3 of its height. These proportions are less extreme in other *Jonkeria* specimens, but still well within the bounds of state ‘1’ for this character (*e.g.*, ~43% of height in SAM-PK-11884).

16. **1**, previously coded as 0. The postfrontal in *Jonkeria* (*e.g.*, TM 212) is somewhat ribbon-like, without discrete differentiation in width between the anterior and posterior portions, but clearly extends posteriorly medial to the frontals.

26. **0**, previously coded as ?. A tabular-paroccipital contact can be seen in FMNH UC 1511.

41. **1**, previously coded as 2. A narrow trough (posteriorly) and interpterygoid vacuity (anteriorly) separating the pterygoids are visible in FMNH UC 1511, SAM-PK-4343, and TM 212. In SAM-PK-11884, the pterygoid quadrate rami appear to be tightly appressed, but this may be attributed to the high level of lateral compression in this specimen.

45. **2**, previously coded as ?. In specimens of *Jonkeria* with reasonably well-preserved occiputs (*e.g.*, FMNH UC 1511), the postparietal consistently appears tall.

54. **0**, previously coded as ?. Several well-preserved, complete mandibles are known for *Jonkeria* (*e.g.*, SAM-PK-12030) and show that no mandibular fenestra is present.

58. **1**, previously coded as 0. A foramen in this position is visible in SAM-PK-12030.

61. **0**, previously coded as 1. *Jonkeria* has five upper incisors (*e.g.*, ROZ.B96).

75. **1**, previously coded as ?. The quadrate in *Jonkeria* specimens (*e.g.*, FMNH UC 1511, SAM-PK-4343) is relatively small compared to that of, *e.g.*, *Dimetrodon*.

78. **1**, previously coded as 0. The lower incisors of *Jonkeria* are subequal in size.

Estemmenosuchus uralensis (PIN 1758/4 [holotype], PIN 1758/22, PIN 1758/25, PIN 1758/79, PIN 1758/300)

8. **2**, previously coded as ?. Although sutural preservation on the dorsal skull surface in mature *Estemmenosuchus* specimens is generally poor, clearer sutures are visible in the less pachyostosed juveniles (*e.g.*, PIN 1758/79, holotype of '*Anoplosuchus tenuirostris*', and PIN 1758/300, holotype of '*Zopherosuchus luceus*'; for synonymy of these taxa, see[40]) and show that a maxilla-nasal-prefrontal contact was present.

12. **1**, previously coded as ?. The specimen PIN 1758/300 shows sutures of the skull roof and the adductor fossa is restricted to the postorbital surface dorsally. Larger (presumed more mature) specimens have proportionally larger temporal fenestrae but the fenestra does not expand further medially onto the skull roof (unlike in anteosaurids), so it is unlikely that adductor musculature extended onto the postfrontal.

13. **1**, previously coded as 0. The anteroposterior width of the postorbital bar in PIN 1758/22 is ~44% of its height.

14. **0**, previously coded as ?. The postorbital is confined to the dorsal margin of the temporal fenestra in PIN 1758/300.

16. **1**, previously coded as 0. A posteromedial extension of the postfrontal is visible in PIN 1758/300.

20. **1**, previously coded as 0. A large, flaring boss in present on the zygoma below the temporal fenestra in *Estemmenosuchus*.

26. **0**, previously coded as ?. A tabular-paroccipital contact is visible in PIN 1758/4.

31. **1**, previously coded as 0. The choanal portion of the vomer terminates in a median ridge as it transitions into the post-choanal portion in *E. uralensis*, visible in PIN 1758/25.

35. **0**, previously coded as ?. The palatines are clearly separated by the post-choanal portion of the vomer in PIN 1758/25.

36. **1**, previously coded as 0. Although numerous, the palatine teeth in *Estemmenosuchus* are confined to a reniform boss (*e.g.*, PIN 1758/25), not spread across the palatine surface.

43. **1**, previously coded as ?. The basipterygoid articulation of *Estemmenosuchus* (*e.g.*, PIN 1758/25) is comparable to that of *Jonkeria*.

44. **1**, previously coded as ?. Teeth are absent on the ectopterygoid of *Estemmenosuchus* (*e.g.*, PIN 1758/25).

45. **0**, previously coded as ?. As seen in PIN 1758/4, the postparietal is wider than it is tall in *E. uralensis*.

53. **0**, previously coded as ?. A distinct coronoid bone is present in *Estemmenosuchus* (*e.g.*, PIN 1758/4). As in *Jonkeria* and *Styracocephalus*, it is an elongate, ribbon-like structure in a roughly horizontal orientation below the postcanine tooth row, overlying the prearticular.

57. **1**, previously coded as 0. A well-developed lateral ridge on the dorsal edge of the surangular is present in *Estemmenosuchus* (*e.g.*, PIN 1758/4).

59. **0**, previously coded as ?. No dorsal articular process is visible in *E. uralensis* specimens preserving the jaw articulation (*e.g.*, PIN 1758/4).

73. **1**, previously coded as 0/1. Expanded muscular attachment on the squamosal within the temporal fenestra is present in *Estemmenosuchus* (*e.g.*, PIN 1758/4).

74. **-**, previously coded as 2. Closure of the interpterygoid vacuity in *Estemmenosuchus* makes this character inapplicable.

75. **1**, previously coded as ?. In *E. uralensis* specimens preserving the quadrate (*e.g.*, PIN 1758/25), the quadrate is substantially lower than half the height of the skull.

77. **1**, previously coded as ?. The occipital elements are preserved in articulation in all specimens of *Estemmenosuchus* in which the entire cranium is not exploded.

78. **1**, previously coded as 0. The lower incisors of *Estemmenosuchus* are subequal in size.

Biseridens qilianicus (IGCAGS V 632 [holotype], IVPP V 12009, IVPP V 16013, IVPP V 22765)

1. **0**, previously coded as ?. The referred skull IVPP V 16013 shows anterior end of snout was oblique, giving it a convex profile.

2. **1**, previously coded as ?. This character is difficult to code in *Biseridens* because both variables change along the length of the snout. In the referred specimen IVPP V 16013, the height and width of the snout are near equal for most of the snout posterior to the approximate mid-point, although width is greater than height close to the orbit and height is greater than width close to the tip of the snout. We coded *Biseridens* as 1 (height and width equal) because it is consistent with the codings for other anomodonts with similar snout proportions in the data matrix.

3. **0**, previously coded as ?. The external naris is immediately posterior to the dorsal process of the premaxilla in IVPP V 16013.

4. **1**, previously coded as ?. The dorsal process of the premaxilla reaches the level of the posterior edge of the canine at the alveolar margin in IVPP V 16013.

5. **1**, previously coded as ?. The premaxillary alveolar margin angles upwards in IVPP V 16013.

7. **1**, previously coded as ?. A short facial process of the septomaxilla is visible in IVPP V 16013.

9. **0**, previously coded as ?. A median boss is absent on the nasals of IVPP V 16013.

13. **1**, previously coded as 0. The original coding for this character was presumably based on the holotype, which has a relatively thin postorbital bar preserved on its left side. However, the referred specimen IVPP V 16013 has notably thicker postorbital bars, which appear better preserved than in the holotype. The length to height ratio is approximately 40% in this specimen, leading to the change in coding for this character.

14. **0**, previously coded as 1. The previous coding for this (posterior process of postorbital extends onto posterior margin of the temporal fenestra) is contradicted by the original (tentative) interpretation of the holotype[41], and is explicitly contradicted by the figures and verbal description of the IVPP V 16013[42]. Our first-hand observations (KDA and CFK) of the specimens agree with the latter interpretation as well.

26. **0**, previously coded as ?. The revised coding is consistent with description in Liu et al. (2010), and is based on the occiput preserved in the holotype.

27. **1**, previously coded as ?. The margins of the choana are well preserved in IVPP V 16013, and they extend well posterior of the incisors.

29. **0**, previously coded as ?. The vomer is paired in IVPP V 16013.

30). **1**, previously coded as ?. The vomer is widest near its mid-length in IVPP V 16013.

31. **1**, previously coded as ?. The posterior part of the the interchoanal portion of the vomer is ridge-like at its junction with the postchoanal portion in IVPP V 16013.

32. **1**, previously coded as ?. The interchoanal portion of the vomer is most trough-like near its posterior end in IVPP V 16013. This morphology is similar to that observed in *Patranomodon*, which is also coded as 1.

33. **1**, previously coded as ?. This region is slightly damaged in IVPP V 16013, but it seems clear that the choanal porition of the vomer was offset ventrally relative to the postchoanal portion.

34. **1**, previously coded as ?. The palatine forms more than 1/3 the length of the lateral margin of the choana in IVPP V 16013.

36. **0**, previously coded as ?. The palatine teeth are large and cover a considerable portion of the ventral surface of the palatine in IVPP V 16013.

38. **0**, previously coded as ?. The holotype of *Biseridens* shows little evidence of denticles on the transverse flange of the palatine, and [41] described them as absent. However, scattered small denticles are present on the transverse flange of IVPP V 16013.

39. **0**, previously coded as ?. IVPP V 16013 demonstrates that the transverse flange of the pterygoid is located under the posterior half of the orbit.

40. **0**, previously coded as ?. Neither the holotype nor IVPP V 16013 are perfectly preserved in this area, but between the two specimens it appears that a shelf posterior to the transverse flange of the pterygoid was absent.

44. **1**, previously coded as ?. The ectopterygoids are imperfectly preserved in IVPP V16013, but no evidence of ectopterygoid teeth is visible in that specimen.

45. **2**, previously coded as ?. Only the holotype is informative for this character, and the occiput of that specimen is highly cracked. However, [41] explicitly stated that the postparietal is taller than wide. Based on our first-hand observations (KDA and CFK) of the specimen, this interpretation seems to be reliable. Indeed, the element could even be slightly wider than suggested by [41] and still be taller than wide.

52. **0**, previously coded as ?. None of the available specimens are perfectly preserved in this area, but IVPP V 16013 and IVPP V 22765 are complete enough indicate that there wasn't a deep posterior incision on the posterior edge of the dentary.

53. **0**, previously coded as ?. A prominent coronoid is visible on the mandible of IVPP V 16013.

54. **0**, previously coded as ?. There is no evidence of a mandibular fesnestra in IVPP V 16013 despite the good preservation of the anterior portion of the angular in the specimen. IVPP V 22765 is more poorly preserved in this area, but still appears to be consistent with this interpretation.

56. **1**, previously coded as ?. Subtle ridges and fossae are present on the reflected lamina of the angular in IVPP V 16013.

57. **?**, previously coded as 0. We do not consider any of the available specimens sufficiently well preserved to code this character.

59. **0**, previously coded as ?. The left articular is preserved in the holotype, and it appears to lack a dorsal process.

60. **1** previously coded as ?. IVPP V 16013 possesses a single upper canine.

61. **0**, previously coded as ?. Liu *et al.*[42] expressed uncertainty about the number of premaxillary teeth but suggested that five were present originally in IVPP V 16013. In our personal observations of the specimen, we found the premaxillary alveoli somewhat damaged, but agree that five is the most likely number of premaxillary teeth, particularly on the right premaxilla.

63. **0**, previously coded as ?. Relatively complete lower incisor crowns are only preserved in IVPP V 22765 and they demonstrate the absence of a heel.

64. **1**, previously coded as ?. Only IVPP V 16013 preserved the premaxilla, but none of the incisor crowns are preserved in that specimen. We assessed this character using the size of

the preserved incisor roots and empty alveoli. Based on these features, the upper incisors appear to have been relatively small, and are seemingly smaller than the preserved postcanine teeth in some cases.

65. **1**, previously coded as ?. There is no evidence of precanine maxillary teeth in IVPP V 16013.

66. **1**, previously coded as ?. IVPP V16013 has a medial fossa anterior to the upper canine to accommodate the lower canine.

67. **0**, previously coded as ?. Upper canines are preserved in IVPP V 16013, and complete or partial lower canines are present in IVPP V 12009, IVPP V 16013, and IVPP V 22765. None show evidence of heels.

68. **0**, previously coded as ?. Based on the positions of preserved teeth and tooth alveoli in IVPP V 16013, there is no evidence of a postcanine diastema in the upper jaw.

70. **0**, previously coded as ?. The upper postcanines are well preserved in the IVPP V 16013 and they are neither triangular nor serrated.

71. **0**, previously coded as ?. The upper postcanines form a continuous row with the canine in IVPP V 16013.

75. **1**, previously coded as ?. The quadrate is only preserved in the holotype, and damage to the occiput makes it difficult to precisely estimate its exact height. However, even a very generous estimate of its size would still imply that its height is less than half of the occiput.

Patranomodon nyaphulii (NMQR 3000 [holotype])

3. **0**, previously coded as 1. The external nares of *Patranomodon* are immediately posterior to the dorsal process of the premaxilla; they as far forward on the snout as is possible.

4. **-**, previously coded as 0. This character is predicated on position of the premaxilla relative to the upper canine, and *Patranomodon* lacks a distinct upper canine.

36. **2**, previously coded as ?. The indentations on the palatine in NMQR 3000 appear to be rugosities rather than alveoli; no tooth crowns are present.

50. **-**, previously coded as 0. No distinct lower canine is present in *Patranomodon*, so the character is inapplicable.

65–68. These characters relate to the canines, which are not distinct tooth positions in *Patranomodon*. As such, these characters have all been coded as inapplicable.

69. **1**, previously coded as ?. A completely literal reading of this character (number of postcanine teeth) would also render it as inapplicable, given that no canine is present, but as it was coded by Liu *et al*.[24] for other taxa without distinct canines, it is coded based on the total number of maxillary teeth (which is still less than 12 in NMQR 3000).

73. **1**, previously coded as 0. Although thin-edged, the temporal fenestra is clearly dissimilar to that of non-therapsid synapsids, and a broad attachment site for jaw musculature is present on the squamosal surface at the posterodorsal edge of the fenestra.

Suminia getmanovi (PIN 2212/10 [holotype], PIN 2212/32, PIN 2212/87)

3. **0**, previously coded as 1. The external nares of *Suminia* are immediately posterior to the dorsal process of the premaxilla; a dinocephalian-like retracted position in the skull profile is an artifact of the elongate premaxillary dentition, not expansion of the premaxilla itself.

4. **-**, previously coded as 0. This character is predicated on position of the premaxilla relative to the upper canine, and *Suminia* lacks a distinct upper canine.

16. **0**, previously coded as ?. One of the skulls preserved in the assemblage of individuals numbered PIN 2212/116 (unassigned skull preserved in right lateral view near the left edge of the block in [43]) preserves sutures in this area well. In that specimen, a narrow process of the parietal wedges between the posterior portion of the postfrontal and the frontal, precluding contact between a posterior extension of the postfrontal and the frontal.

30. **1**, previously coded as ?. The vomer is visible in PIN 2212/33 and in one of the skulls preserved in the assemblage of individuals numbered PIN 2212/116 (unassigned specimen at upper right corner of block in [43]).

31. **1**, previously coded as ?. The posteriormost preserved portion of the vomer in one of the skulls preserved in the assemblage of individuals numbered PIN 2212/116 (unassigned specimen at upper right corner of block in [43]) is suggestive of the presence of a median ridge in this area.

32. **1**, previously coded as ?. PIN 2212/33 clearly shows the presence of a trough on the ventral surface.

36. **2**, previously coded as ?. The palatine is exposed and reasonably well preserved in PIN 2212/87 and shows that it was edentulous.

50. **-**, previously coded as 0. No distinct lower canine is present in *Ulemica*, so the character is inapplicable.

65–68. These characters relate to the canines, which are not distinct tooth positions in *Suminia*. As such, these characters have all been coded as inapplicable.

Gorgonops torvus (AMNH FARB 5515, BP/1/4089, NHMUK PV R1647 [holotype], SAM-PK-2343, SAM-PK-K11180)

13. **1**, previously coded as 0. An anteroposteriorly-expanded postorbital bar is characteristic of *Gorgonops*, particularly at the ventral base of the bar. In BP/1/4089, for example, the anteroposterior width (measured at mid-height) of the bar is ~70% of its height.

16. **1**, previously coded as 0. The postfrontal curves posteromedially in *Gorgonops* (*e.g.*, BP/1/4089, NHMUK PV R1647), with a weakly tapering posterior process medial to the frontal.

42. **0**, previously coded as 1. The median parasphenoid blade present in most gorgonopsian taxa is not the same as the widely-separated parasagittal ridges of the basicranial rami of the pterygoid in burnetiamorphs.

58. **0**, previously coded as ?. No foramen in this location can be observed in, *e.g.*, BP/1/4089.

71. **0**, previously coded as ?. The upper postcanine tooth row of *Gorgonops* is clearly not confluent with the incisor tooth row (see, *e.g.*, SAM-PK-K11180).

74. **1**, previously coded as 1/2. Theriodonts represent an exception to the inapplicability of this character in cases where the basicranial rami of the pterygoids are tightly appressed posterior to the transverse processes, because the interpterygoid vacuity in these taxa (when it occurs) is present anterior (also sometimes extending medial) to the transverse processes. Closure of the interpterygoid vacuity over the course of ontogeny occurs in many theriodont taxa; in *Gorgonops* few of the very large specimens have well-prepared palates. An open, albeit small, interpterygoid vacuity is present in, *e.g.*, BP/1/4089 and SAM-PK-K11180.

78. **1**, previously coded as 0. The lower incisors of *Gorgonops* are subequal in size.

Lycaenops ornatus (AMNH FARB 2240 [holotype], CGS FL 17)

16. **1**, previously coded as 0. The postfrontal morphology of *Lycaenops* (*e.g.*, AMNH FARB 2240) is similar to that of *Gorgonops*.

17. **0**, previously coded as 2. The pineal foramen of *L. ornatus* is not situated in a tall, chimney-like boss as in a biarmosuchian or dinocephalian; the surrounding portions of the parietals are either flat (although this may be exaggerated by overpreparation) or only very weakly raised at the edge of the foramen (*e.g.*, AMNH FARB 2240).

27–34. The genus *Lycaenops* is in need of revision; the numerous species referred to this genus by [10] do not appear to form a monophylum (CFK, pers. obs.) Here, coding is based only on the type species *L. ornatus*; unfortunately, no definite *L. ornatus* specimens have the anterior palate exposed, so all characters related to the choana and vomer have been recoded as **?**.

36. **1**, previously coded as 2. Palatine teeth are present in *L. ornatus* (*e.g.*, AMNH FARB 2240) and are set on a raised boss, as in other gorgonopsians.

37. **0**, previously coded as 1. Teeth are present on the palatal exposure of the pterygoid in a small patch posteromedial to the palatine in AMNH FARB 2240.

38. **0**, previously coded as 1. A tooth row is present on the transverse process of the pterygoid in *L. ornatus* (*e.g.*, AMNH FARB 2240).

39. **1**, previously coded as 2. The transverse process of the pterygoid is located under the anterior half of the orbit in *L. ornatus* (*e.g.*, AMNH FARB 2240).

42. **0**, previously coded as 1. A median parasphenoid blade, not parasagittal pterygoid ridges, is present in *L. ornatus* (*e.g.*, AMNH FARB 2240).

46. **1**, previously coded as 0. The occiput in *L. ornatus* is essentially vertical (*e.g.*, AMNH FARB 2240), unlike in *Gorgonops* in which it is angled anterodorsally such that most of the occiput is visible in dorsal view of the skull.

74. **-**, previously coded as 2. Closure of the interpterygoid vacuity in *Lycaenops* makes this character inapplicable.

Cyonosaurus longiceps (BP/1/137, FMNH UC 1515 [holotype], NHMUK PV R15981, SAM-PK-10054, SAM-PK-K5511, TM 2040, TMM 42442-1)

16. **1**, previously coded as 0. The postfrontal morphology of *Cyonosaurus* (*e.g.*, FMNH UC 1515) is similar to that of *Gorgonops* and *Lycaenops*, but with an even more elongated posterior portion.

17. **0**, previously coded as 2. The pineal foramen of *Cyonosaurus* is not situated in a tall, chimney-like boss as in a biarmosuchian or dinocephalian; the surrounding portions of the parietals are either flat (although this may be exaggerated by overpreparation) or only very weakly raised at the edge of the foramen (*e.g.*, TMM 42552-1).

38. **0**, previously coded as 1. Although small and often lost due to damage or overpreparation, teeth are present at least medially on the transverse process of the pterygoid in *Cyonosaurus* specimens preserving this feature (*e.g.*, TM 2040).

42. **0**, previously coded as 1. A median parasphenoid blade, not parasagittal pterygoid ridges, is present in *Cyonosaurus* (*e.g.*, NHMUK PV R15981).

66. **0**, previously coded as ?. A precanine embayment in the choana accommodates the lower canine in *Cyonosaurus* (*e.g.*, NHMUK PV R15981), as in all other gorgonopsians.

74. **1**, previously coded as 1/2. A small, thin interpterygoid vacuity is present anteromedial to the transverse processes of the pterygoids in, *e.g.*, BP/1/137.

75. **1**, previously coded as ?. The quadrate of *Cyonosaurus* (preserved in, *e.g.*, TM 2040) is a relatively small bone, less than half the height of the skull.

76. **1**, previously coded as ?. The epipterygoid of *Cyonosaurus* (preserved in, *e.g.*, SAM-PK-K5511) is similar to that of other gorgonopsians, with an elongate, narrow columella and a horizontally-oriented footplate at the level of the primary palate.

78. **1**, previously coded as ?. The lower incisors of *Cyonosaurus* are subequal in size.

Supplementary Table 1. Bayes factor compares and across clock models for this dataset.

**Clock model**	**Marginal lnL**	**BF**
IGR	−1057.1	0
ILN	−1058.11	2.02
WN (IGR of v.<3.2.8)	−1064.96	15.72
TK02	−1064.26	14.32

Supplementary Fig. 6 (next page)**. Comparison of evolutionary trees and divergence times for the major groups of therapsids using distinct tree calibration strategies. a,** Maximum compatible tree from the tip-dating only analysis. **b**, Results from the tip + node dating analysis (including the Mallorcan site age data). Node values represent median ages; purple error bars represent the 95% highest posterior density (HPD) intervals; branch thickness proportional to posterior probabilities; dotted lines connect equivalent notes between trees.

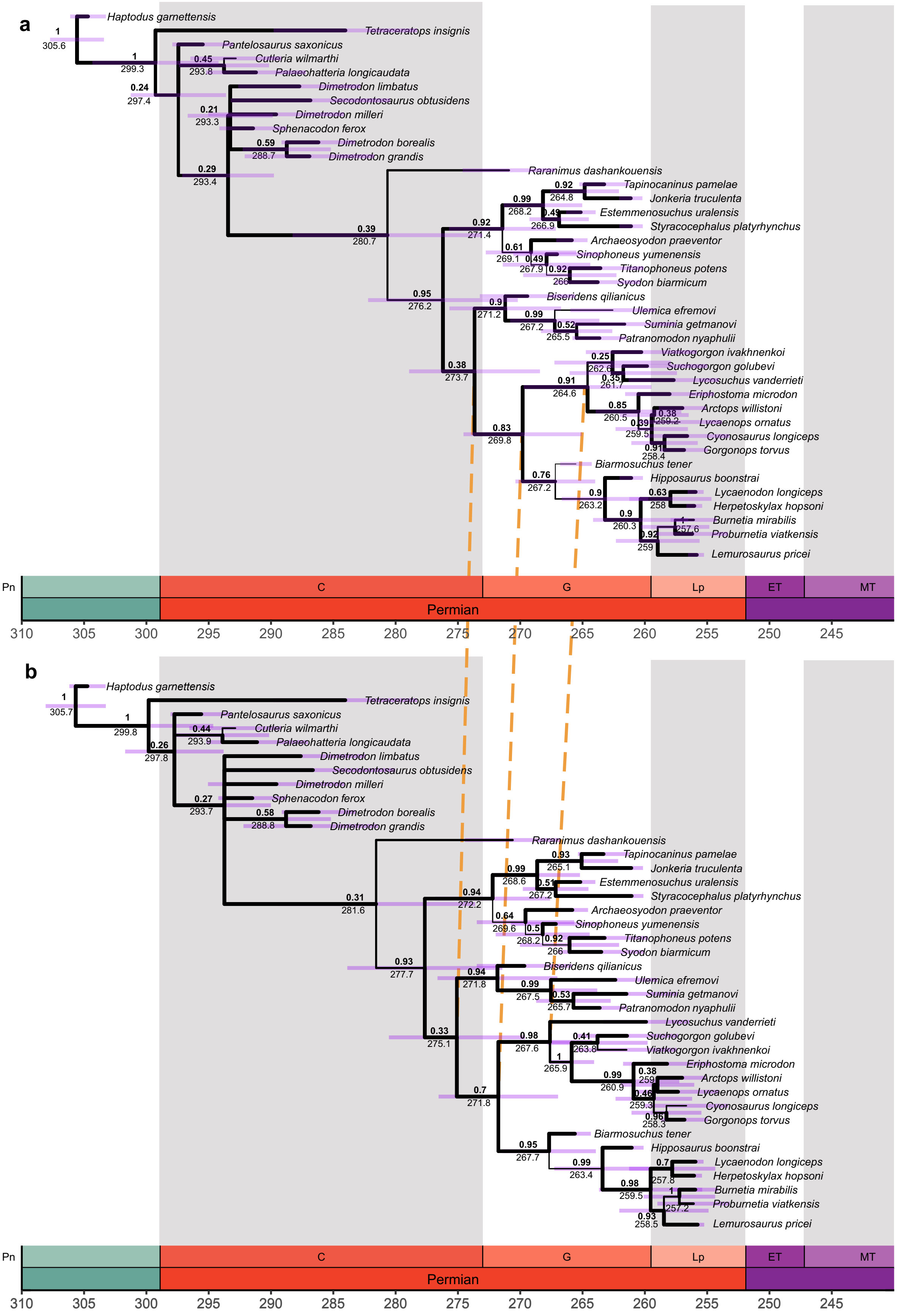
a
Haptodus garnettensis
Tetraceratops insignis
Pantelosaurus saxonicus
Cutleria wilmarthi
Palaeohatteria longicaudata
Dimetrodon limbatus
Secodontosaurus obtusidens
Dimetrodon milleri
Sphenacodon ferox
Dimetrodon borealis
Dimetrodon grandis
Raranimus dashankouensis
Tapinocaninus pamelae
Jonkeria truculenta
Estemmenosuchus uralensis
Styracocephalus platyrhynchus
Archaeosyodon praeventor
Sinophoneus yumenensis
Titanophoneus potens
Syodon biarmicum
Biseridens qilianicus
Ulemica efremovi
Suminia getmanovi
Patranomodon nyaphulii
Viatkogorgon ivakhnenkoi
Suchogorgon golubevi
Lycosuchus vanderrieti
Eriphostoma microdon
Arctops willistoni
Lycaenops ornatus
Cyonosaurus longiceps
Gorgonops torvus
Biarmosuchus tener
Hipposaurus boonstrai
Lycaenodon longiceps
Herpetoskylax hopsoni
Burnetia mirabilis
Proburnetia viatkensis
Lemurosaurus pricei
Pn
C
G
Lp
ET
MT
Permian
310
305
300
295
290
285
280
275
270
265
260
255
250
245
b
Haptodus garnettensis
Tetraceratops insignis
Pantelosaurus saxonicus
Cutleria wilmarthi
Palaeohatteria longicaudata
Dimetrodon limbatus
Secodontosaurus obtusidens
Dimetrodon milleri
Sphenacodon ferox
Dimetrodon borealis
Dimetrodon grandis
Raranimus dashankouensis
Tapinocaninus pamelae
Jonkeria truculenta
Estemmenosuchus uralensis
Styracocephalus platyrhynchus
Archaeosyodon praeventor
Sinophoneus yumenensis
Titanophoneus potens
Syodon biarmicum
Biseridens qilianicus
Ulemica efremovi
Suminia getmanovi
Patranomodon nyaphulii
Lycosuchus vanderrieti
Suchogorgon golubevi
Viatkogorgon ivakhnenkoi
Eriphostoma microdon
Arctops willistoni
Lycaenops ornatus
Cyonosaurus longiceps
Gorgonops torvus
Biarmosuchus tener
Hipposaurus boonstrai
Lycaenodon longiceps
Herpetoskylax hopsoni
Burnetia mirabilis
Proburnetia viatkensis
Lemurosaurus pricei
Pn
C
G
Lp
ET
MT
Permian
310
305
300
295
290
285
280
275
270
265
260
255
250
245

Supplementary References

1. Kammerer, C. F. & Masyutin, V. Gorgonopsian therapsids (*Nochnitsa* gen. nov. and *Viatkogorgon*) from the Permian Kotelnich locality of Russia. *PeerJ* **6**, e4954 (2018).

2. Hopson, J. A. & Barghusen, H. B. in *The Ecology and Biology of the Mammal-Like Reptiles* (eds Hotton, N. III, MacLean, P. D., Roth, J. J. & Roth, E. C.) 83–106 (Smithsonian Institution Press, 1986).

3. Sigogneau-Russell, D. *Handbuch der Paläoherpetologie / Encyclopedia of Paleoherpetology. Teil 17B/I / Part 17B/I: Theriodontia I. Phthinosuchia, Biarmosuchia, Eotitanosuchia, Gorgonopsia* (Gustav Fischer Verlag, 1989).

4. Kemp, T. S. On the functional morphology of the gorgonopsid skull. *Philos Tr Roy Soc B* **256**, 1–83. (1969).

5. Bendel, E.-M., Kammerer, C. F., Kardjilov, N., Fernandez, V. & Fröbisch, J. Cranial anatomy of the gorgonopsian *Cynariops robustus* based on CT-reconstruction. *PLoS ONE* **13**, e0207367 (2018).

6. Kammerer, C. F. Systematics of the Rubidgeinae (Therapsida: Gorgonopsia). *PeerJ* **4**, e1608 (2016).

7. Kammerer, C. F. Anatomy and relationships of the South African gorgonopsian *Arctops* (Therapsida, Theriodontia). *Papers in Palaeontol* **3**, 583–611 (2017).

8. Kammerer, C. F. Cranial osteology of *Arctognathus curvimola*, a short-snouted gorgonopsian from the Late Permian of South Africa. *Papers in Palaeontol* **1**, 41–58 (2015).

9. Kammerer, C. H., Smith, R. M. H., Day, M. O. & Rubidge, B. S. New information on the morphology and stratigraphic range of the mid-Permian gorgonopsian *Eriphostoma microdon* Broom, 1911. *Papers in Palaeontol* **1**, 201–221 (2015).

10. Sigogneau, D. *Révision systématique des gorgonopsiens sud-africains* (Centre National de la Recherche Scientifique, 1970).

11. Araújo, R., Fernandez, V., Polcyn, M. J., Fröbisch, J. & Martins, R. M. S. Aspects of gorgonopsian paleobiology and evolution: insights from the basicranium, occiput, osseous labyrinth, vasculature, and neuroanatomy. *PeerJ* **5**, e3119 (2017).

12. King, G. M. *Handbuch der Paläoherpetologie / Encyclopedia of Paleoherpetology. Teil 17C / Part 17C: Anomodontia*. (Gustav Fischer Verlag, 1988).

13. Broom, R. Catalogue of types and figured specimens of fossil vertebrates in the American Museum of Natural History; ii Permian, Triassic and Jurassic reptiles of South Africa. *Bull Am Mus Nat Hist* **25**, 105–164 (1915).

14. Tatarinov, L. P. A postcranial skeleton of the gorgonopian *Viatkogorgon ivachnenkoi* (Reptilia, Theriodontia) from the Upper Permian Kotelnich locality, Kirov Region. *Paleontol J* **38**, 437–447 (2005).

15. Gebauer, E. V. I. in *Early Evolutionary History of the Synapsida* (eds Kammerer, C. F., Angielczyk, K. D. & Fröbisch, J.) 185–207 (Springer, 2014).

16. Colbert, E. H. The mammal-like reptile *Lycaenops*. *Bull Am Mus Nat Hist* **89**, 353–404 (1948).

17. Blob, R. W., Huttenlocker, A. K., Kammerer, C. F. & Sidor, C. A. Comparative allometry of femoral curvature in gorgonopsian versus therocephalian therapsids. *In*: Meeting Program and Abstracts, SVP 75th Annual Meeting, Society of Vertebrate Paleontology: p. 91 (2015).

18. Romer, A. S. *Osteology of the Reptiles* (University of Chicago Press, 1956).

19. Hopson, J. A. Patterns of evolution in the manus and pes of non-mammalian therapsids. *J Vertebr Paleontol* **15**, 615–639 (1995).

20. Sidor, C. A. New information on gorgonopsian pedal morphology based on articulated material from Zambia. *J Afr Earth Sci* **191**, 104533 (2022).

21. Schaeffer, B. The morphological and functional evolution of the tarsus in amphibians and reptiles. *Bull Am Mus Nat Hist* **78**, 395–472 (1941).

22. Kümmell, S. B. & Frey, E. Range of movement in ray I of manus and pes and the prehensility of the autopodia in early Permian to Late Cretaceous non-anomodont Synapsida. *PLoS ONE* **9**, e113911 (2014).

23. Kümmell, S. B. & Frey, E. Digital arcade in the autopodia of Synapsida: Standard position of the digits and dorsoventral excursion angle of digital joints in the rays II–V. *Palaeobiodiv Palaeoenvir* **92**, 171–196 (2012).

24. Liu, J., Rubidge, B. & Li, J. New basal synapsid supports Laurasian origin for therapsids. *Acta Palaeontol Pol* **54(3)**, 393–400 (2009).

25. Amson, E. & Laurin, M. On the affinities of *Tetraceratops insignis*, an Early Permian synapsid. *Acta Palaeontol Pol* **56**, 301–312 (2011).

26. Eberth, D. A. The skull of *Sphenacodon ferocior*, and comparisons with other sphenacodontines (Reptilia: Pelycosauria). *New Mexico Bureau of Mines and Mineral Resources Circular* **190**, 1–39 (1985).

27. Reisz, R. R., Berman, D. S. & Scott, D. The cranial anatomy and relationships of *Secodontosaurus*, an unsusual mammal-like reptile (Synapsida: Sphenacodontidae) from the early Permian of Texas. *Zool J Linn Soc* **104**, 127-184 (1992).

28. Brink, K. S., Maddin, H. C., Evans, D. C. & Reisz, R. R. Re-evaluation of the historic Canadian fossil *Bathygnathus borealis* from the Early Permian of Prince Edward Island. *Can J Earth Sci* **52(12)**, 1109–1120 (2015).

29. Laurin, M. & Reisz, R. R. *Tetraceratops* is the oldest known therapsid. *Nature* **345**, 249–250 (1990).

30. Laurin, M. & Reisz, R. R. The osteology and relationships of *Tetraceratops insignis*, the oldest known therapsid. *J Vertebr Paleontol* **16**, 95–102 (1996).

31. Conrad, J. & Sidor, C. A. Re-evaluation of *Tetraceratops insignis* (Synapsida: Sphenacodontia). *J Vertebr Paleontol* **31(abstract volume)**, 42A (2001).

32. Spindler, F. The skull of *Tetraceratops insignis* (Synapsida, Sphenacodontia). *Palaeovertebrata* **43**, 1–11 (2020).

33. Duhamel, A., Benoit, J., Rubidge, B. S. & Liu, J. A re-assessment of the oldest therapsid *Raranimus* confirms its status as a basal member of the clade and fills Olson's gap. *The Science of Nature* **108**, 26 (2021).

34. Ivakhnenko, M. F. Biarmosuches from the Ocher faunal assemblage of eastern Europe. *Paleontol J* **33**, 289–296 (1999).

35. Hopson, J. A. in *Origins of the Higher Groups of Tetrapods: Controversy and Consensus Systematics of the nonmammalian Synapsida and implications for patterns of evolution in synapsids* (eds Schultze, H.-P. & Trueb, L.) 635–693 (Comstock Publishing Associates, 1991).

36. Grine, F. E. Dinocephalians are not anomodonts. *J Vertebr Paleontol* **17**, 177–183 (1997).

37. Orlov, J. A. [Predatory dinocephalians from the Isheevo Fauna (titanosuchians)]. *Trudy Paleontologicheskogo Instituta, Akademiya Nauk SSSR* **72**, 1–114 (1958) [in Russian].

38. Kammerer, C. F. Systematics of the Anteosauria (Therapsida: Dinocephalia). *J Syst Palaeontol* **9**, 261–304 (2011).

39. Liu, J. Osteology, ontogeny, and phylogenetic position of *Sinophoneus yumenensis* (Therapsida, Dinocephalia) from the middle Permian Dashankou Fauna of China. *J Vertebr Paleontol* **33(6)**, 1394–1407 (2013).

40. Ivakhnenko, M. F. Eotherapsids from the East European Placket (Late Permian). *Paleontol J* **37**, S339–S465 (2003).

41. Li, J. & Cheng, Z. First discovery of an eotitanosuchian (Therapsida, Synapsida) of China. *Vertebrata PalAsiatica* **35**, 268–282 (1997).

42. Liu, J., Rubidge, B. & Li, J. A new specimen of *Biseridens qilianicus* indicates its phylogenetic position as the most basal anomodont. *Proc Roy Soc B* **277**, 285–292 (2010).

43. Fröbisch, J. & Reisz, R. R. The postcranial anatomy of *Suminia gemanovi* (Synapsida: Anomodontia), the earliest known arboreal tetrapod. *Zool J Linn Soc* **162**, 661–698 (2011).
