# Supplementary material for: Early–middle Permian Mediterranean gorgonopsian suggests an equatorial origin of therapsids
Source: Nat Commun. 2024 Dec 17;15:10346. doi: 10.1038/s41467-024-54425-5 (PMC11652623; doi:10.1038/s41467-024-54425-5)
Supplement: Supplementary file 3 — Reporting Summary [file 41467_2024_54425_MOESM3_ESM.pdf]

nature portfolio

Corresponding author(s): Rafel Matamales Andreu

Last updated by author(s): 10/10/2024

Reporting Summary

Nature Portfolio wishes to improve the reproducibility of the work that we publish. This form provides structure for consistency and transparency in reporting. For further information on Nature Portfolio policies, see our Editorial Policies and the Editorial Policy Checklist.

Statistics

For all statistical analyses, confirm that the following items are present in the figure legend, table legend, main text, or Methods section.

n/a	Confirmed	
☐	☒	The exact sample size (*n*) for each experimental group/condition, given as a discrete number and unit of measurement
☒	☐	A statement on whether measurements were taken from distinct samples or whether the same sample was measured repeatedly
☒	☐	The statistical test(s) used AND whether they are one- or two-sided<br>*Only common tests should be described solely by name; describe more complex techniques in the Methods section.*
☒	☐	A description of all covariates tested
☒	☐	A description of any assumptions or corrections, such as tests of normality and adjustment for multiple comparisons
☐	☒	A full description of the statistical parameters including central tendency (e.g. means) or other basic estimates (e.g. regression coefficient) AND variation (e.g. standard deviation) or associated estimates of uncertainty (e.g. confidence intervals)
☒	☐	For null hypothesis testing, the test statistic (e.g. *F*, *t*, *r*) with confidence intervals, effect sizes, degrees of freedom and *P* value noted<br>*Give P values as exact values whenever suitable.*
☐	☒	For Bayesian analysis, information on the choice of priors and Markov chain Monte Carlo settings
☒	☐	For hierarchical and complex designs, identification of the appropriate level for tests and full reporting of outcomes
☒	☐	Estimates of effect sizes (e.g. Cohen's *d*, Pearson's *r*), indicating how they were calculated

Our web collection on statistics for biologists contains articles on many of the points above.

Software and code

Policy information about availability of computer code

Data collection	No software was used for data collection.
Data analysis	Our data was compiled in Mesquite (v. 3.04); our analysis were performed in the phylogenetic software TNT (v 1.5) and Mr. Bayes (v. 3.2.8). All the necessary code for phylogenetic analyses are provided as Supplementary Data.

For manuscripts utilizing custom algorithms or software that are central to the research but not yet described in published literature, software must be made available to editors and reviewers. We strongly encourage code deposition in a community repository (e.g. GitHub). See the Nature Portfolio guidelines for submitting code & software for further information.

Data

Policy information about availability of data

All manuscripts must include a data availability statement. This statement should provide the following information, where applicable:

- Accession codes, unique identifiers, or web links for publicly available datasets
- A description of any restrictions on data availability
- For clinical datasets or third party data, please ensure that the statement adheres to our policy

All data generated and analysed are freely available online as Supplementary Data at: https://doi.org/10.7910/DVN/9RNJAE

Research involving human participants, their data, or biological material

Policy information about studies with human participants or human data. See also policy information about sex, gender (identity/presentation), and sexual orientation and race, ethnicity and racism.

Reporting on sex and gender	Not applicable.
Reporting on race, ethnicity, or other socially relevant groupings	Not applicable.
Population characteristics	Not applicable.
Recruitment	Not applicable.
Ethics oversight	Not applicable.

Note that full information on the approval of the study protocol must also be provided in the manuscript.

Field-specific reporting

Please select the one below that is the best fit for your research. If you are not sure, read the appropriate sections before making your selection.

☐ Life sciences ☐ Behavioural & social sciences ☒ Ecological, evolutionary & environmental sciences

For a reference copy of the document with all sections, see nature.com/documents/nr-reporting-summary-flat.pdf

Ecological, evolutionary & environmental sciences study design

All studies must disclose on these points even when the disclosure is negative.

Study description	We provide an anatomical description of a new fossil specimen showing the main characters of the therapsid clade Gorgonopsia. We use its proposed age to calibrate a Bayesian relaxed morphological clock analysis that quantitatively estimates the origin of the major therapsid clades for the first time, using an updated and expanded phylogenetic dataset. We use these results to discuss the evolution and palaeobiogeography of Permian non-mammalian therapsids.
Research sample	A partial skeleton, its elements were found scattered in a single stratigraphic bed.
Sampling strategy	Not applicable.
Data collection	The specimen was collected by RM-A, ÀG and JF, using mechanical methods such as buzzsaws, hammers and chisels. It was consolidated in situ with Paraloid B72, and extracted in the form of large blocks, as the bones are very brittle. The spatial and stratigraphic position of all the elements was recorded with digital pictures and a notebook compiling all the necessary information. The specimens were later prepared at the Preparation Lab at the ICP (Cerdanyola del Vallès), using airscribes. The prepared bones were examined directly by CFK and RM-A.
Timing and spatial scale	The bones were collected in the field seasons of 2019 and 2021, from a surface of about 0.6 square meters.
Data exclusions	No data were excluded.
Reproducibility	All data necessary to verify our observations have been deposited online (see data availability statement) and all necessary input data files to reproduce analytical protocols have also been deposited online (see code availability statement).
Randomization	Not applicable.
Blinding	Blinding was not relevant to this data set, and set of analyses.

Did the study involve field work? ☒ Yes ☐ No

Field work, collection and transport

Field conditions	The fossil deposit is located at the base of the cliffs of the northwestern coast of Mallorca (Balearic Islands, western Mediterranean). Excavations were carried out in autumn or spring, when there is usually no precipitation and temperatures are around 20°C.
Location	Torrent de na Nadala 2, the studied fossil deposit, is at about 1 m above the sea level, and its coordinates are: 39°40'20"N 2°29'22"E.

Access & import/export	The site was accessed on foot, using a dangerous, narrow path that descends to the cliff base. Permits for excavation and preparation of the specimen were granted by the Comissió de Patrimoni Històric del Consell Insular de Mallorca (reference numbers 306/2019 and 75/2021 for excavation, and 374/2020 and 619/2020 for preparation).
Disturbance	No disturbance was caused by the present study.

Reporting for specific materials, systems and methods

We require information from authors about some types of materials, experimental systems and methods used in many studies. Here, indicate whether each material, system or method listed is relevant to your study. If you are not sure if a list item applies to your research, read the appropriate section before selecting a response.

Materials & experimental systems

n/a	Involved in the study
☒	☐ Antibodies
☒	☐ Eukaryotic cell lines
☐	☒ Palaeontology and archaeology
☒	☐ Animals and other organisms
☒	☐ Clinical data
☒	☐ Dual use research of concern
☒	☐ Plants

Methods

n/a	Involved in the study
☒	☐ ChIP-seq
☒	☐ Flow cytometry
☒	☐ MRI-based neuroimaging

Palaeontology and Archaeology

Specimen provenance	The studied specimen was collected from the Torrent de na Nadala 2 site (Banyalbufar, Mallorca, Balearic Islands, western Mediterranean), with coordinates 39°40'20"N 2°29'22"E. Permits for excavation and preparation of the specimen were granted by the Comissió de Patrimoni Històric del Consell Insular de Mallorca (reference numbers 306/2019 and 75/2021 for excavation, and 374/2020 and 619/2020 for preparation).
Specimen deposition	The specimen is currently deposited at the Museu de Mallorca (Palma, Mallorca, Balearic Islands, Spain).
Dating methods	The dating information was extracted from Matamales-Andreu et al. (2022), see references.

☒ Tick this box to confirm that the raw and calibrated dates are available in the paper or in Supplementary Information.

Ethics oversight	The study protocol was approved by the Comissió de Patrimoni Històric del Consell Insular de Mallorca.

Note that full information on the approval of the study protocol must also be provided in the manuscript.

Plants

Seed stocks	Not applicable.
Novel plant genotypes	Not applicable.
Authentication	Not applicable.
